# A Motion Capturing and Energy Harvesting Hybridized Lower‐Limb System for Rehabilitation and Sports Applications

**DOI:** 10.1002/advs.202101834

**Published:** 2021-08-19

**Authors:** Shan Gao, Tianyiyi He, Zixuan Zhang, Hongrui Ao, Hongyuan Jiang, Chengkuo Lee

**Affiliations:** ^1^ School of Mechatronics Engineering Harbin Institute of Technology Harbin 150001 China; ^2^ Department of Electrical and Computer Engineering National University of Singapore 4 Engineering Drive 3 Singapore 117583 Singapore; ^3^ Center for Intelligent Sensors and MEMS (CISM) National University of Singapore 4 Engineering Drive 3 Singapore 117583 Singapore; ^4^ NUS Graduate School for Integrative Science and Engineering National University of Singapore Singapore 117456 Singapore

**Keywords:** hybridized lower‐limb system, piezoelectric energy harvester, rehabilitation, sports monitor, triboelectric sensors

## Abstract

Lower‐limb motion monitoring is highly desired in various application scenarios ranging from rehabilitation to sports training. However, there still lacks a cost‐effective, energy‐saving, and computational complexity‐reducing solution for this specific demand. Here, a motion capturing and energy harvesting hybridized lower‐limb (MC‐EH‐HL) system with 3D printing is demonstrated. It enables low‐frequency biomechanical energy harvesting with a sliding block‐rail piezoelectric generator (S‐PEG) and lower‐limb motion sensing with a ratchet‐based triboelectric nanogenerator (R‐TENG). A unique S‐PEG is proposed with particularly designed mechanical structures to convert lower‐limb 3D motion into 1D linear sliding on the rail. On the one hand, high output power is achieved with the S‐PEG working at a very low frequency, which realizes self‐sustainable systems for wireless sensing under the Internet of Things framework. On the other hand, the R‐TENG gives rise to digitalized triboelectric output, matching the rotation angles to the pulse numbers. Additional physical parameters can be estimated to enrich the sensory dimension. Accordingly, demonstrative rehabilitation, human‐machine interfacing in virtual reality, and sports monitoring are presented. This developed hybridized system exhibits an economic and energy‐efficient solution to support the need for lower‐limb motion tracking in various scenarios, paving the way for self‐sustainable multidimensional motion tracking systems in near future.

## Graphical abstract

A motion capturing and energy harvesting hybridized lower‐limb system is proposed for low‐frequency biomechanical energy harvesting and lower‐limb motion sensing. Combining with the Internet of Things framework, the hybridized system exhibits an economic and energy‐efficient solution to support the need for lower‐limb motion tracking in various applicable scenarios, paving the way for self‐sustainable multidimensional body motion tracking systems in near future.

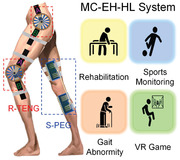

## 1 Introduction

Wearable electronics have experienced blooming development and advancement in the past few years,^[^ ^1^, ^2^, ^3^ ^]^ offering diversified functionalities ranging from physical sensing^[^ ^4^, ^5^ ^]^ to chemical sensing^[^ ^6^, ^7^ ^]^ for various applications, such as healthcare monitoring,^[^ ^8^, ^9^, ^10^ ^]^ rehabilitation,^[^ ^11^, ^12^ ^]^ disease diagnosis/treatment,^[^ ^13^, ^14^, ^15^ ^]^ and sports instruction/training.^[^ ^16^, ^17^ ^]^ For instance, various wearable sensors attached to the skin or worn on the body have been developed for gait and posture monitoring,^[^ ^18^, ^19^, ^20^ ^]^ which have emerged as promising candidates for stroke or Parkinson's disease (PD) monitoring.^[^ ^21^, ^22^, ^23^ ^]^ Such wearable sensors also hold great promise for sports applications by assisting the athletes and coaches in analyzing personal body mechanics,^[^ ^24^, ^25^ ^]^ which will help adjust techniques to avoid injury or reduce effort. Under the thriving Internet of Things (IoT) framework,^[^ ^26^, ^27^, ^28^ ^]^ the sensory information could be uploaded to the cloud for analysis and visualization, which may be further used to construct digital entities in the virtual reality (VR) or augmented reality (AR) world for more advanced applications.^[^ ^29^, ^30^, ^31^, ^32^ ^]^ Generally, wearable sensing technologies could be categorized into flexible/stretchable devices^[^ ^33^, ^34^, ^35^ ^]^ and rigid component‐constructed designs.^[^ ^36^, ^37^, ^38^ ^]^ Flexible sensors provide excellent conformability to the skin but with limited sensing accuracy and consistency, while rigid structures mostly contribute to a superior sensing precision with a sacrifice in deformability.

Targeting for healthcare, rehabilitation, and sports monitoring, body motion tracking is of vital importance. One of the widely used techniques for body motion monitoring is image processing, where a camera is in need of capturing numerous images for further analysis.^[^ ^39^ ^]^ However, the video‐based motion tracking system has high requirements for lighting, and the recognition results will be significantly affected by the illuminance condition and the camera position. Meanwhile, the massive amount of images would pose a severe threat to the privacy of the user. Another common technique is the inertial measurement unit (IMU) which integrates accelerometers and gyroscopes to provide an estimation of an object's orientation and acceleration in space.^[^ ^40^, ^41^ ^]^ In spite of its good accuracy and reliability, the IMU suffers from a large amount of data per second, accumulated error, and complicated data analytics. The cost of the aforementioned technologies is also high, which largely limits their widespread use for practical applications. Alternatively, wearable sensors then have emerged as promising candidates for body motion tracking to avoid the privacy issue and reduce the computation cost.^[^ ^42^, ^43^ ^]^

Among the widely investigated sensing mechanisms, self‐powered sensors based on triboelectric nanogenerators (TENGs) and piezoelectric generators (PEGs) stand out as a promising technology to reduce systematic energy consumption.^[^ ^44^, ^45^, ^46^, ^47^ ^]^ Various designs of TENG and PEG on different wearable platforms have been developed for diversified applications, including vital sign monitoring,^[^ ^48^, ^49^, ^50^ ^]^ body motion sensing,^[^ ^51^, ^52^, ^53^, ^54^ ^]^ chemical sensing,^[^ ^55^, ^56^, ^57^ ^]^, and human‐machine interfacing toward VR/AR applications.^[^ ^58^, ^59^, ^60^, ^61^ ^]^ Meanwhile, TENGs and PEGs also experienced flourishing advancement in implantable applications such as energy harvesting,^[^ ^62^, ^63^, ^64^ ^]^ medical treatment,^[^ ^65^, ^66^, ^67^ ^]^ in vivo sensing,^[^ ^68^, ^69^ ^]^ and rehabilitation.^[^ ^70^, ^71^, ^72^ ^]^ Hybridized devices combining the characteristics and advantages of the two prominent mechanisms have also been thoroughly investigated to further extend the systematic performance and capability in divergent applications.^[^ ^73^, ^74^, ^75^, ^76^ ^]^

Typically, lower‐limb rehabilitation is desperately in need of a wearable mobile machine, i.e., exoskeleton, to support and assist the lower‐limb motion of the patients suffering from impaired ambulation.^[^ ^77^, ^78^ ^]^ Due to the high requirement on structural strength and endurance, the exoskeletons are mostly constructed with rigid mechanical components that wrap around the lower limb and designed with movable joints to allow for the free movement of the lower limb.^[^ ^79^, ^80^ ^]^ In this particular scenario, wearable sensors consisting of hard mechanical components are highly preferred to provide accurate motion tracking, which is readily integrable with the exoskeleton frames.^[^ ^81^, ^82^ ^]^ Utilizing the low‐cost 3D printing technique and minimalist sensor design, wearable sensors in robust mechanical structures can be developed for body motion sensing, with a reduced computational cost and satisfactory recognition precision.^[^ ^83^, ^84^ ^]^ Such wearable sensors with simplified configuration and data processing could outperform flexible sensors, aiming at lower‐limb gesture monitoring for existing exoskeleton systems in the near future.^[^ ^85^, ^86^ ^]^

**Figure 1:**
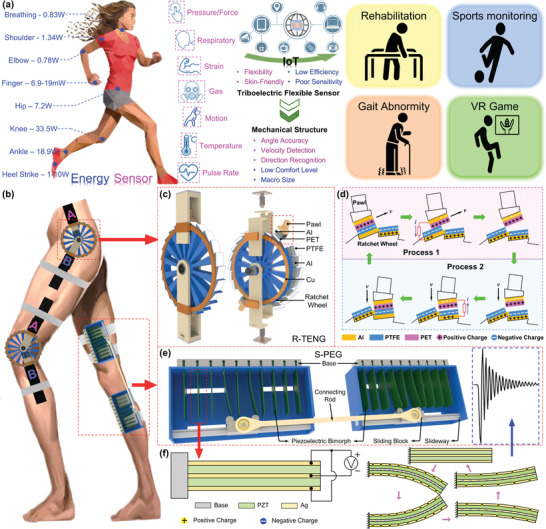
The MC–EH–HL system for lower‐limb motion sensing and low‐frequency biomechanical energy harvesting. a) Schematic diagram showing the available power from body motions on different segments^[^ ^94^ ^]^ (assuming an 80 kg person was walking at a frequency of 1 Hz per cycle, and the walking speed of ≈4 km h^−1^), the characteristic comparison of the flexible sensors and rigid‐structure‐based sensors, and the future applications. The woman model is reproduced with permission from VectorStock.com (https://www.vectorstock.com). b) Configuration of the MC–EH–HL system, which consists of two main components: R‐TENG and S‐PEG. The leg model is reproduced with permission from ARTSTATION.com (https://www.artstation.com). c) Schematic diagram and explosive view of the R‐TENG. d) Working principle of a single unit of the R‐TENG under rotation mode. e) Schematic diagram of the S‐PEG component. f) Working principle of the S‐PEG under vibration mode.

For the long‐term operation of wearable sensors, a reliable and sustainable power source becomes one of the critical challenges.^[^ ^87^, ^88^, ^89^, ^90^ ^]^ Battery, which though is the widespread solution, is restricted by drawbacks of limited lifespan, periodic replacement, possible hazards to human health, and long‐term reliability. Mechanical energy harvesters that convert waste biomechanical energy into electrical power have shed light on the possible clean energy sources for wearable systems.^[^ ^91^, ^92^, ^93^ ^]^ In particular, lower‐limb motions produce the highest power compared to other body parts, as indicated in **Figure** **1**a.^[^ ^94^ ^]^ The general conversion mechanisms include electromagnetic, piezoelectric, and triboelectric. Electromagnetic energy harvesters are characterized by high output current and low output voltage under high‐frequency vibrational conditions.^[^ ^95^, ^96^, ^97^ ^]^ Considering the low‐frequency pattern of the lower‐limb movement, piezoelectric^[^ ^98^, ^99^, ^100^ ^]^ and triboelectric^[^ ^101^, ^102^, ^103^, ^104^ ^]^‐based energy harvesters and self‐powered sensors are profoundly desired to scavenge waste energy and monitor the limb gestures.

Herein, a 3D‐printed motion capturing and energy harvesting hybridized lower‐limb (MC–EH–HL) system is proposed to realize low‐frequency biomechanical energy harvesting and lower‐limb motion monitoring. The MC–EH–HL system is achieved by integrating an efficient sliding block–rail‐based piezoelectric bimorph array generator (S‐PEG) for energy scavenging and a minimalist ratchet‐based triboelectric nanogenerator (R‐TENG) for bidirectional joint rotation detection. The unique sliding‐block–rail structure enables the integration of overall 20 piezoelectric bimorphs on one limb, leading to a high‐power density and charging speed even at low operating frequencies. A modified ratchet–pawl structure is leveraged to recognize joint rotation direction, angle, and speed, in order to quantify the lower‐limb motions detailing to all movable joints. On the basis of joint rotation detection, extra physical parameters related to the lower‐limb postures and dynamics can be estimated through kinetic analysis, including raised foot height, step length, and kicking force. With these features, the R‐TENG can serve as a wearable assistive device for gait abnormity detection, exercise monitoring, and lower‐extremity capacity detection for rehabilitation applications. The feasibility of R‐TENG for human‐machine interface (HMI) in a VR treasure hunting game and a VR sports training program is further investigated. On top of that, a self‐sustained IoT sensing system can be attainable with the S‐PEG converting lower‐limb kinetic energy into electricity. In general, the proposed MC–EH–HL system offers a low‐cost and energy‐efficient platform for lower‐limb motion tracking and multiple kinetic parameter estimation without extra sensors, which would be highly desired in applications of interest, such as rehabilitation and sports monitoring.

## 2 Structure Design of the MC–EH–HL System

To adaptably fit the wearable devices with human body lower limbs to scavenge kinetic energy and monitor all kinds of human motion, an MC–EH–HL system with energy harvesting and sensing functionalities is developed by integrating S‐PEG and R‐TENG, as shown in Figure 1b. To precisely monitor the joint rotation angle of the lower limb, a mechanical structure would be preferred to ensure a stable and reliable sensing output, especially considering the existence of the rigid mechanical frame of the conventional exoskeletons for gait‐impaired patients. Specifically, bidirectional rotation sensing is required for comprehensive joint rotation monitoring, which has been rarely developed and reported before.^[^ ^105^, ^106^, ^107^ ^]^ Ratchet is one of the typical mechanical structures, which is characterized by a set of angled teeth in which a pawl engages, allowing the motion in one direction only. The ratchet mechanism can not only perform the contact–separation and lateral sliding procedure during rotation but also process multiple unique advantages such as simple construction, convenient manufacture, operational reliability, unidirectional, and intermittent movement. Inspired by the delicate ratchet mechanism, we have designed a bidirectional rotation sensor based on the triboelectric elements to collect the motion signals during rotation, that is, the R‐TENG.

An R‐TENG is composed of a ratchet wheel and two ratchet pawls made by low‐cost 3D printing, connected with coaxial bearings in cooperation to enable the bidirectional rotation, as shown in Figure 1c. The ratchet structure is designed with detailed structural dimensions as listed in Table S1 (Supporting Information), which owns 24‐wheel teeth with a width of 10 mm and a maximum diameter of 100 mm. Thus, each tooth of the wheel represents 15° when the pawls sweep forward. As the ratchet pawl is required to engage the tooth of the wheel, it is designed in a right‐angled trapezoid shape at the side view. Furthermore, a cylindrical‐shaped hollow part is fixed on the pawl in the longitude direction to form an up‐and‐down moving unit between the spring and wheel teeth. Both pawls are pressed to the wheel tightly under the preforce from springs, with polyethylene terephthalate (PET) and polytetrafluoroethylene (PTFE) thin films attached to the surface of the pawls and wheel, respectively. Figure S1a (Supporting Information) describes the detailed dimension diagram for the R‐TENG structure. By setting free the rotary direction of the ratchet wheel and the cooperation with two ratchet pawls, both pawls can rotate clockwise or counterclockwise with regard to the centra of the wheel. The engagement between the pawl and teeth only allows the pawl to sweep forward along the wheel teeth, which will be fixed to the wheel in the opposite rotation direction. As one of the pawls (e.g., pawl A) is controlled to rotate clockwise, it will move forward along the wheel that is fixed by pawl B; while in the counterclockwise direction, the wheel along with pawl A rotates together, in which case the pawl B sweeps forward against wheel teeth even though it is not moving (see Figure S2 in the Supporting Information). In other words, in an arbitrary rotation motion of one pawl, one of the pawls functions as the driving pawl that moves forward along the wheel teeth, while the other one serves as the locking pawl that involves no relative motion to the wheel. In this way, the opposite rotary directions result in the different participation of the components, leading to the direction‐recognition functionality through two channels from two pawls, which realizes a bidirectional rotation to match up the actions of lower‐limb joints. In a rotary process illustrated in Figure 1d, the first half cycle involves the lateral sliding of the pawl on the engaged tooth that generates a small positive voltage in the output signal until the pawl slides away (Process 1). Subsequently, the pawl is rapidly pressed on the next tooth with a high negative output due to its large spring force (Process 2). As the R‐TENG experiences clockwise or counterclockwise rotations, there will be the corresponding sensing signals generated in one of the dual channels from the driving pawl, hence allowing easy differentiation on the rotation direction. Meanwhile, the rotation angle as a multiple of 15° can be accurately detected by the digitized triboelectric output, which is in virtue of the intermediate sliding/contacting motions of the driving pawl.

To endow the system with self‐sustainable sensing capability, the S‐PEG comprising the lead zirconate titanate (PZT) bimorph is proposed, as shown in Figure 1e, which is the well‐known structure in piezoelectric energy harvesting technology due to its superiors including simple structure, large output, and high efficiency. Previously reported piezoelectric‐based energy harvesters are mostly focusing on enlarging the deflection of the bimorphs to increase the output power or broadening the frequency band for adapting to more working scenarios.^[^ ^108^, ^109^, ^110^ ^]^ In this work, an interconnected sliding block–rail structure is introduced to the energy harvesting system for the first time not only aiming at improving the output performance but also supporting a prolonged output duration in the sliding motions. Moreover, moving forward to the biomechanical energy harvesting from the human lower limb, this structure is beneficial in adapting to the relative varying angles between thigh and shank during various motions.

As shown in Figure 1e, ten pieces of PZT bimorphs of an array are parallelly inserted into the base structure with one free end sitting in a frame. A ball‐bearing‐based sliding block equipped with two identical half‐cylinders is able to slide along the sliding rail. The half‐cylinders work as the plectrum due to their symmetrical circular contact with the sharp tip of bimorphs in order to offer a smooth bending force during motion. The bending force affects the bending deflection of the PZT bimorphs and ultimately is reflected in the voltage output performance, which is controllable by the overlapping area between bimorph and cylinder (see Figure S3 in the Supporting Information). Two frames are integrated using a connecting rod configured with bearing at both ends, which conforms to a linked device and can be easily tied around the thigh and shank. In fact, the length of the connection rod imposes a significant impact on the smoothness of motion conduction and the optimal position of two frames on the lower limb, which further affects the participation of bimorphs and output. Hence, the calculative process of these two parameters is demonstrated in Figure S4 (Supporting Information). The detailed dimensions of the S‐PEG are illustrated in Figure S1b (Supporting Information). It can be seen in the schematic diagram of S‐PEG, four bimorphs are deflected simultaneously when the sliding block shifts on the sliding rail. Accordingly, four contemporaneous voltage outputs will be generated and they can be accumulated to boost up overall output performance. In addition, the cumulated voltage is characterized with a wide time scope due to the consecutive deflections of four bimorphs, resulting in a more sustainable power generation capacity. Through this way, any relative motions of thigh and shank would be transferred into bearing rotation accompanied by forces applied to the sliding block, which contributes to the continuous shift of each block along its sliding rail. In other words, the S‐PEG device is capable of transforming 3D lower‐limb motion into 2D sliding–bending movement in the planar frame, which is then converted to 1D bimorph oscillation, that is, the harvesting process from kinetic energy to electricity. The structural diagram of a single bimorph with its working mechanism is depicted in Figure 1f. As the bending deflection increases, more charges are accumulated on the silver electrodes. Once the bimorph is set free, it oscillates at its resonant frequency, and then the vibration tapers off, as shown in the waveform highlighted in the blue inset.

## 3 Characterization of the MC–EH–HL System

**Figure 2:**
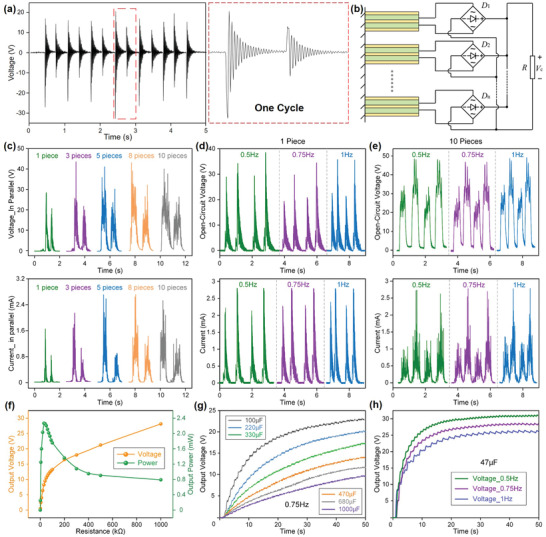
The basic characterization of the S‐PEG. a) Open‐circuit voltage of a single PZT bimorph. The enlarged graph shows the oscillating voltage in one cycle. b) Circuit diagram of the in‐parallel connection for multiple PZT bimorphs. c) Open‐circuit voltage and short‐circuit current of different pieces of bimorphs in parallel. The open‐circuit voltage and short‐circuit current with two repeated cycles of d) a single bimorph and e) ten bimorphs’ array excited at different frequencies. Regarding the power curve and charge performance, 20 bimorphs in the S‐PEG are tested as shown in f) the output voltage under various loads and the power curve of the S‐PEG, g) charging curve of different capacitors (100/220/330/470/680/1 mF) with the S‐PEG attached to the lower limb and operated under 0.75 Hz walking frequency, h) charging curves of a 47 µF capacitor under different walking frequencies (0.5/0.75/1 Hz).

To look into the performance and characteristics of the MC–EH–HL system in terms of energy harvesting and rotation sensing, the S‐PEG and R‐TENG are tested individually. A single bimorph PZT is characterized first by plucking it with the sliding block back and forth in the whole movable range of the frame, and its corresponding open‐circuit voltage in multiple cycles is provided in **Figure** **2**a. As explained previously, the PZT bimorphs will be deflected and then released to free vibration at their resonance frequencies when the sliding block moves, which contributes to the oscillating voltage spikes for each deflection. Meanwhile, it can be observed that the output voltage of deflection in one direction is larger than the reversed direction, which is attributed to the slightly inclined PZT bimorph from the perpendicular base direction originated from the manufacturing deviation. As demonstrated in the red dashed, a series of higher outputs are accompanied by lower ones, which also reflects the moving direction of the slide blocks, i.e., first in the anti‐inclined direction followed by the reversed sliding motion. Figure S5a (Supporting Information) lists the rectified output voltages of ten individual bimorphs, where observable discrepancies between them are measured presumably due to the manufacturing and installation deviations. Here, ten pieces of PZT bimorphs are integrated into each 3D‐printed mechanical frame to boost up the output voltage by virtue of the large area of the thigh and shank sides. Theoretically, the PZT bimorphs can be connected in series or in parallel to increase their voltage or current output. Correspondingly, the parallel‐connected PZT bimorphs with the circuit diagram provided in Figure 2b generate higher output currents while producing lower voltages than the series‐connected ones, as shown in Figure 2c and Figure S5b,c (Supporting Information). As the number of bimorphs increases, it can be observed that both output current and voltage are mostly broadened in the time scope; in the meantime, the absolute amplitudes of some peaks are improved, resulting from the overlapping of few random peaks during the sliding motions. Since the charging capability is of vital importance for practical applications, the PZT bimorphs in series or parallel are used to charge a 47 µF capacitor through a conventional bridge rectifier for comparison, and the charging curves are provided in Figure S5d (Supporting Information). It can be observed that the parallel‐connected bimorphs contribute to a higher charging voltage presumably due to the higher output current, which hence is adopted for the following testing. Figure 2d,e presents the output voltages and currents of a single bimorph and ten bimorphs under different sliding frequencies. The outputs from ten bimorphs are characterized by more spikes evenly distributed in each sliding cycle with slightly improved output amplitudes. It is also interesting to be noted that the sliding frequency imposes a negligible effect on the outputs for both single bimorph and ten bimorphs. This is attributed to the unique structure design where the oscillating output is solely related to the bimorph's deflection, which is determined by the structural parameters and irrelevant with the sliding frequency. Considering the low‐frequency characteristic of human motions, the S‐PEG is a promising candidate to scavenge waste energy effectively from various lower‐limb dynamics. The maximum current can reach 2.8 mA in these tests, which also implies an excellent energy conversion capability of the S‐PEG.

To investigate the output power of the S‐PEG, its output voltage on different loads is measured, and the corresponding output powers are calculated, as shown in Figure 2f. Maximum power of 2.4 mW is achieved with 50 kΩ load resistance under 0.75 Hz block sliding. Previously, a similar piezoelectric knee‐joint energy harvester based on rotary PZT bimorph array is developed to harvest human lower‐limb motion by Zhu and co‐workers.^[^ ^111^, ^112^, ^113^ ^]^ With four pieces of PZT bimorphs embedded, the peak output power density reaches 13.94 µW cm^−3^ or 13.40 µW g^−1^ at 0.9 Hz. At a lower frequency of 0.75 Hz,^[^ ^114^ ^]^ our S‐PEG has achieved a high peak output power density of 22.004 µW cm^−3^ or 16.861 µW g^−1^, which is attributed to the dense PZT bimorph arrangement and the unique interconnected sliding block configuration. Besides, the attachment of S‐PEG on the side of thigh and shank enables a larger size, giving rise to a higher overall output power during low‐frequency lower‐limb motions. Since the energy generated from body motions needs to be stored for further use, Figure 2g,h shows the charging curves of capacitors with varying capacitances under multiple working frequencies. Under a frequency of 0.75 Hz, the charging curves of capacitors with the capacitance of 100 µF, 220 µF, 330 µF, 470 µF, 680 µF, and 1 mF are illustrated in Figure 2g. The charging speed is gradually reducing as the capacitances of capacitors are getting larger. The charged voltage of the 100 µF capacitor can reach up to 23.5 V in just 50 s, with a calculated charging rate of 47 µC s^−1^ (calculated by Δ*Q* = *C*Δ*V*). Similarly, the voltage can be charged up to 8 V in 50 s for the 1 mF capacitor but with a slower charging rate, that is, 160 µC s^−1^. The charging rate is not only affected by the capacitances but also related a lot to the operating frequencies, which corresponds to the bimorph sliding speeds. Basic frequencies of 0.5, 0.75, and 1 Hz are adopted to charge a 47 µF capacitor, with the charging curves depicted in Figure 2h. Since a higher frequency provides more times of activation of the PZT bimorphs in a certain period, it necessarily concludes in faster charging speed and higher output. Hence, in 40 s, the charged voltage can reach up to 30 V with 1 Hz operating frequency, while reduces to 25 V at 0.5 Hz. More comparisons of the charging speed with the reported energy harvesters based on different mechanisms are listed in Table S2 (Supporting Information). The excellent output performance of the S‐PEG device under low operating frequencies (smaller than 1 Hz) is particularly suitable for energy harvesting from lower‐limb motions, which can be widely employed as a prominent power source or auxiliary battery to supply the wearable electronics or wireless transmission modules for diversified applications.

**Figure 3:**
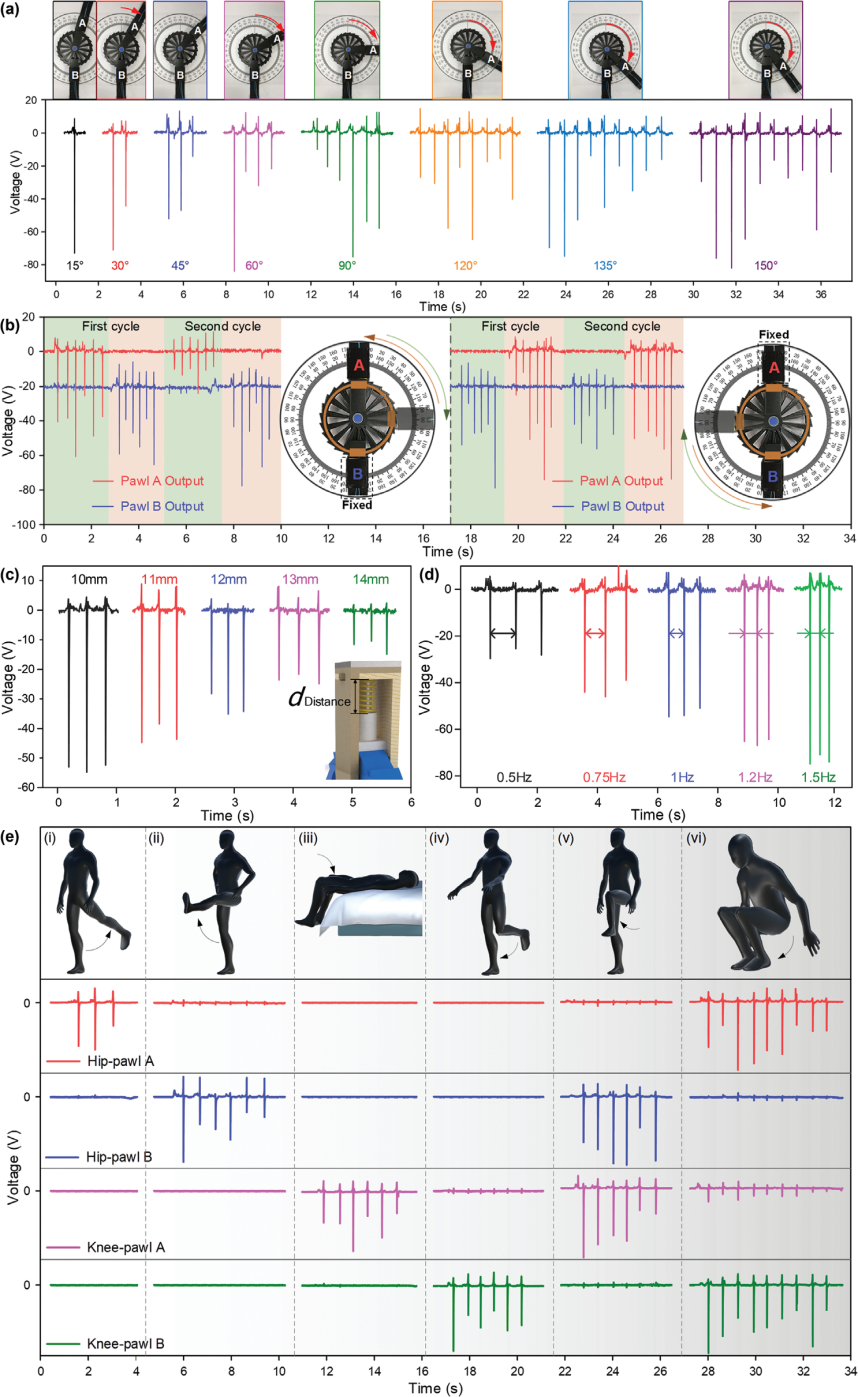
The basic characterization of the R‐TENG. a) The output voltage of the R‐TENG as it is rotated at different degrees in the clockwise direction. b) The output voltages of the R‐TENG as the pawl A (left) or the pawl B (right) is controlled to rotate clockwise or counterclockwise 90° for two cycles, with the other pawl fixed during rotation. The initial angle between pawl A and pawl B is 90°. c) The output voltages of a single unit with various preset forces. d) Output voltages under different rotating speeds. e) The output voltages of two R‐TENGs located at knee and hip on the left leg of user as shown in the virtual figure under various lower‐limb motions: (i) thigh backward kicking, (ii) thigh forward kicking, (iii) lying down with shank bending, (iv) shank backward kicking, (v) knee lifting, and (vi) squatting.

Though flexible or stretchable triboelectric sensors for lower‐limb motion capturing have been vastly developed for their excellent wearable comfortability, the limited accuracy and the susceptibility to environmental interferences largely prohibits them from practical applications. Aiming at lower‐limb rehabilitation applications where a rigid exoskeleton is mostly indispensable, the 3D‐printed R‐TENG based on the ratchet mechanism has been proposed to detect rotatory angle, speed, and direction with high sensitivity and precision. First, the one‐directional rotation angle detection of the R‐TENG ranging from 15° to 150° is tested, as shown in **Figure** **3**a. In the experimental setup, pawls A and B are posited in line with an angulometer background to visualize the changes of rotary angles in real‐time. Because of the mechanical interference between two pawls, the rotary angle in one direction is maximized at 150°. Here, pawl B is fixed, while pawl A is functioning as the driving pawl; hence, the pulse‐like output voltage is generated in the pawl‐A channel. Since each tooth on the wheel represents 15° when the pawl sweeps forward, the rotary angles can be measured as a multiple of 15° by the numbers of the voltage peaks. Compared to the triboelectric sensors using signal amplitude as the sensing parameter, this digitalized output feature is highly robust and anti‐interference against environmental noises and changes (Figure S6, Supporting Information), contributing to a precise and stable sensory performance. Similarly, the digitized current output can be detected, as shown in Figure S7a (Supporting Information). Figure S7b (Supporting Information) provides photos of the R‐TENG attached to the knee joint at different rotation angles, where pawl A is fixed to the thigh, and pawl B is fixed to the shank. To demonstrate the bidirectional rotation sensing capability of the R‐TENG, one of the pawls is fixed, while the other pawl is the freely movable end, which is controlled to rotate clockwise or counterclockwise for 90° (Figure 3b). It can be observed that only one channel generates effective output voltages during an arbitrary rotation, which corresponds to the driving pawl and the rotation direction. For instance, as pawl B is fixed and pawl A is controlled to rotate clockwise, pawl A will sweep forward along the wheel teeth, generating pulsed output in channel pawl A. In the reversed direction, pawl A rotates with the wheel, causing sweeping forward in pawl B along the wheel teeth, hence contributing to outputs in channel pawl B. In other words, pulses in the pawl‐A channel indicate clockwise rotation, and pawl B corresponds to the reversed direction, i.e., counterclockwise. Meanwhile, six voltage spikes can be detected for each rotation cycle, which is in accordance with the rotation angle (90°). In this way, both rotating direction and angle can be monitored through the two‐channel output.

As a critical component in the R‐TENG, the influence of the preforce stored with the pawl's spring on the triboelectric output is also investigated. Figure 3c shows the output voltage under 45° rotation with ascending preset forces, which are controlled by the operating distance of the spring (its original length is 15 mm). It can be seen that smaller distances give rise to higher outputs, contributed by the larger preset forces. However, the resistance force during rotating will also be increased with higher preset spring forces. In consideration of the large output and the smooth operation of the R‐TENG, an optimal spring distance of 12 mm was chosen. Apart from the preset force, a higher rotation speed also contributes to a larger output, as depicted in Figure 3d. In the meantime, the time duration between the spikes is also a precise reflection of the rotation speed.

To deduct the dynamics of the lower limb, it is vital to monitor the rotation angle of both knee joint and hip joint. With two R‐TENGs attached to the knee and hip joints with the ratchet wheels set in the clockwise direction of wheel teeth, as shown in Figure 1b, the lower‐limb motions can be monitored. The same arrangement is adopted for the following tests without further illustration. Figure 3e depicts the output signals of the user performing six different types of motions, i.e., thigh backward kicking, thigh forward kicking, lying down with shank bending, shank backward kicking, knee lifting, and squatting. Different types of motions can be easily distinguished based on the voltage peaks of multichannel. Motions from (i) to (iv) only trigger one channel and the angles are measured as 45° and 90°. Photos showing the user wearing the R‐TENG and performing the postures in Figure 3e(i), (ii), (iv) are provided in Figure S8 (Supporting Information). Moving to more complex motions as (v) and (vi), two sensors are triggered simultaneously during the movements. It should be noted that the number of pulses indicates the angle variations between the shank and thigh or the thigh and upper body, and the channel from pawl A or pawl B implies the motion dynamics during this bending movement. For example, in Figure 3e(iii), (iv), both knee bending angles are 90°, which can be calculated from the pulsed output in channel A or B. Meanwhile, the output from pawl A or pawl B indicates the driving pawl, which further suggests that the bending motion is caused by the movement of the thigh or shank. This multidimensional sensing information lays the foundation for future practical applications such as rehabilitation and sports monitoring.

## 4 Self‐Sustained IoT Sensing

**Figure 4:**
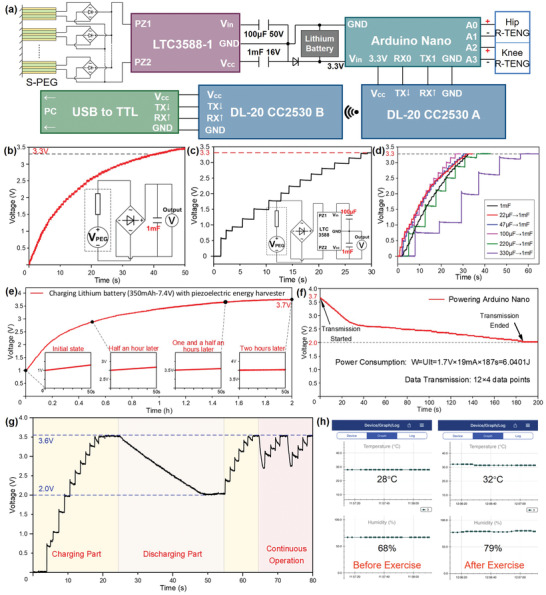
Self‐sustainable IoT sensing. a) Circuit design of the self‐sustained IoT sensing system. b) Conventional charging curve on the 1 mF capacitor of the S‐PEG. The inset shows the circuit diagram for the S‐PEG. c) Charging curves through LTC3588‐1 on the 1 mF capacitor. The inset shows the circuit diagram S‐PEG and LTC3588‐1. d) Charging curves through LTC3588‐1 with different small capacitors (22/47/100/220/330 µF) to charge 1 mF. e) Lithium battery charging performance with S‐PEG. The insets are the enlarged charging curve at different stages. f) Voltage drops as the battery is connected to the Arduino Nano. g) Charging and discharging curve of the Bluetooth module with S‐PEG. h) Enlarged screenshot on a smartphone showing the collected temperature and humidity attached to the forearm skin surface: (i) before exercise, (ii) after exercise.

The upsurge of the number of sensor nodes in the IoT network is desperately in need of sustainable energy sources to further extend the lifetime of IoT sensors. Energy harvester, which converts available renewable ambient energy into usable electricity, has become a promising candidate in the realization of self‐sustainable IoT sensors. In this work, the energy conversion S‐PEG can function as the power source for the R‐TENG, forming the MC–EH–HL system as described in **Figure** **4**a, with an electronic components setup shown in Figure S9 (Supporting Information). S‐PEG is equipped on the lower limb of the human body to scavenge kinetic energy during various motions, e.g., walking. Its functionality here is regarded as an auxiliary battery working along with the lithium battery to supply the IoT sensing system, which contains the R‐TENG, the microprogrammed control unit (MCU) Arduino Nano, radio‐frequency (RF) modules of DL‐20 CC2530 A and B, and the interface converter of USB to TTL. Lower‐limb motions are captured and transferred by different channels of R‐TENGs into electrical data and then separately acquired by Arduino Nano. Afterward, Arduino delivers the signals to the RF modules: the transmitting terminal A and the receiving terminal B. The real‐time data are further sent to a personal computer with a USB to TTL serial port for further analysis and processing.

A power management circuit is introduced to bridge the S‐PEG and MCU, which processes a power management chip of LTC3588‐1 and a lithium battery. To explore the performance of the power management unit, the charging curves of a 1 mF capacitor with S‐PEG attached to the lower limb under a walking frequency of 0.75 Hz have been investigated. Figure 4b depicts the charging curve through a conventional bridge rectifier, where the 1 mF capacitor can be charged up to 3.3 V (the Arduino Nano threshold) in around 40 s, which shows a slow and gentle charging rate. As depicted in Figure 4c, the LTC3588‐1 chip is employed accompanied by two capacitors with capacitances of 100 µF and 1 mF. Its functionality of power management reflects in a level‐to‐level charging mode, which allows charges to initially accumulate on an input capacitor with smaller capacitance (100 µF) and then transfer the total chargers to fill in a larger capacitor (1 mF). Because larger capacitors lead to higher inevitable charge loss compared with smaller ones, this level‐to‐level charging mode would be beneficial for charging rate increment. Hence, a 30% speedup on the charging rate can be obtained through LTC3588‐1, presenting a shortened charging time (28 s) to reach 3.3 V. Due to the specific charging scheme of this power management circuit, the selection of the smaller capacitor connected to the LTC3588‐1 is also critical. As shown in Figure 4d, we have tested the charging performance over the 1 mF capacitor with a series of small capacitors, whose capacitances range from 22 to 330 µF. It can be observed that capacitances smaller than 100 µF generate faster charging rates than directly connecting the 1 mF with LTC3588‐1. However, large capacitances such as 330 µF reduce the charging speed instead. Thus, the combination of 100 µF – 1 mF exhibits an optimum performance on the charging rate, which is used in the following tests.

Considering the large power consumption of the Arduino Nano board, a lithium battery (350 mAh–7.4 V) is required with the auxiliary power supply from S‐PEG. Figure 4e shows the charging curve of the lithium battery‐powered with S‐PEG at a frequency of 0.75 Hz. The battery with a capacity of 350 mAh can be charged from 1.0 to 3.7 V within 2 h. The four insets demonstrate the charging states at different timings from a fast growth rate of the initial state to the steady output of 3.7 V two hours later. The Arduino Nano is turned on when the voltage is higher than the threshold (3.3 V). Figure 4f depicts the voltage drop of the battery when it is connected to an Arduino Nano for power supply. Due to high power loss for initiating the operation process, a sharp decline occurs in the beginning and then steadily reduces to the minimum working voltage. The effective operation duration lasts 180 s for maintaining Arduino at its operating state, which consumes about 6.0401 J energy. As wireless transmission consumes a large amount of power, the Arduino Nano connected with the RF module only works within a short period with the same energy level, successfully transmitting 12 sets of four‐channel data.

As what has been demonstrated, the MC–EH–HL system can be functionalized as a wearable self‐sustained sensing system for human motion detection and wireless transmission. It not only offers the feasibility to achieve a self‐powered system but also provides a potential for wirelessly sending SOS alarm in emergency circumstances, e.g., falling down. In the long term, the self‐sustained MC–EH–HL system could achieve real‐time monitoring with long endurance in diversified healthcare applications.

Besides directly employing S‐PEG's output for battery charging and subsequent MCU powering, the functionality of the self‐sustained IoT system could be further broadened by storing the scavenged kinetic energy to power additional healthcare monitoring sensors. The temperature and humidity of the body surface certainly reflect the physical health condition to some extent, which has proven that the variation could be an assistance for medical diagnose and prediction. For the elderly and patients, as well as infants and young children, extreme situations including too high/low temperature and humidity may imply severe diseases. Besides, detailed physical monitoring for users may benefit from the records of temperature and humidity after daily training and sports events to preliminarily understand the subject's physical conditions. Accordingly, a self‐sustained IoT sensing system for temperature and humidity monitoring is built, as shown in Figure S10 (Supporting Information). The Bluetooth module incorporated with a humidity and temperature sensor is powered by the S‐PEG with the assistance of the LTC3588‐1 chip. To present the charging performance in a daily life scenario, the S‐PEG is tested under the knee bending frequency of 0.75 Hz. Figure 4g shows the voltage on the energy storage capacitor in three operation scenarios, i.e., capacitor charging through the moving S‐PEG (energy non‐sufficient for powering the Bluetooth module), discharging to the Bluetooth module (no input from the S‐PEG), and continuous operation (simultaneous charging and discharging). During the charging process, the capacitor can be charged up to 3.6 V within 20 s, which then consumes energy for sensing and wireless transmission with a slow voltage drop from 3.6 to 2 V in 25 s. During this time, 58 data points are sent out to the smartphone with accurate transmitting time and temperature/humidity values. Once the stored energy is enough for initiating the operation of the Bluetooth module and the S‐PEG keeps working on harvesting energy from lower‐limb motions, a continuous operation mode can be achieved where the temperature and humidity values are continuously monitored. To demonstrate the healthcare monitoring functionality of the temperature/humidity sensor, it was attached to the bare skin of the upper arm to monitor the physical conditions during exercise. Increases of both temperature and humidity level have been observed after 20 min exercise, which reflects the heating up of body temperature and the perspire phenomena on the skin surface (Figure 4h). Looking forward, by incorporating low‐power sensors with other functionalities of interest, self‐sustainable IoT sensors with broadened applications are feasible in near future.

## 5 Task‐Specific Monitoring for Rehabilitation Applications

**Figure 5:**
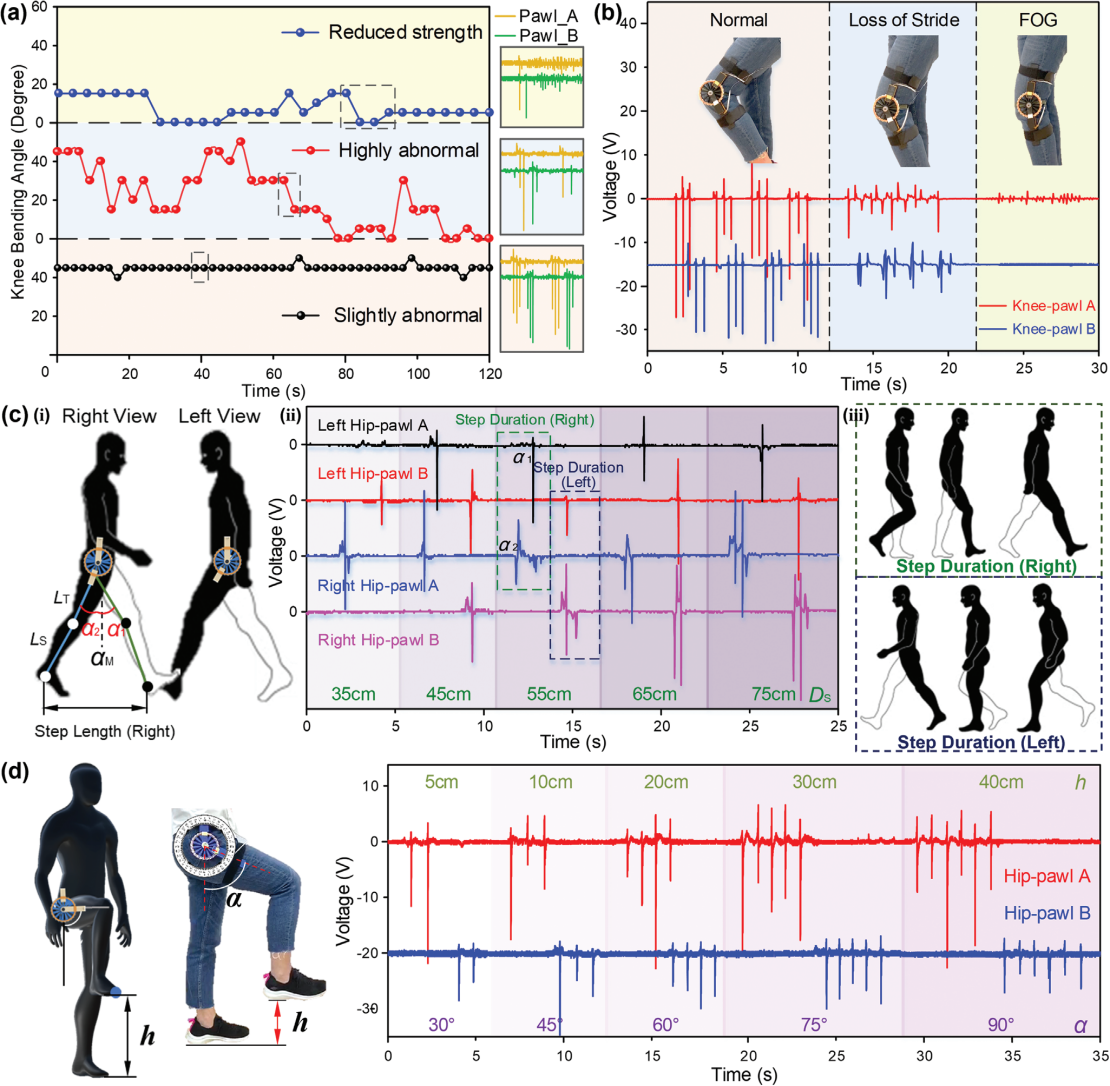
Lower‐limb motion monitoring with R‐TENGs for rehabilitation applications. a) The detected rotation angles through the R‐TENG on knee joint during a series marching on the spot motions, with three imitated gait features for future clinical diagnosis (slightly abnormal, highly abnormal, and reduced strength). Three insets are provided to show the raw data from the R‐TENG. b) Voltage of the R‐TENG on the knee as the user is stepping to monitor typical parkinsonian gaits (normal, loss of stride, and freezing of gait period). c) Output voltage corresponds to different step lengths in a full gait cycle (ii), where (i) demonstrates right and left view with R‐TENGs equipped on each hip and (iii) presents the step duration of a gait cycle.^[^ ^116^ ^]^ d) Voltage signals of the R‐TENG on hip with different heights of foot.

Disorders of gait or posture are debilitating and common for the elderly and mobility‐impaired patients. Adequate and precise recognition of these disorders is critical as they can provide useful hints to the underlying pathology such as neurological disorders in early courses.^[^ ^115^ ^]^ More importantly, lower‐limb posture recognition is vital for rehabilitation by offering timely feedback to the exercise and physical therapy interventions. The R‐TENG that can detect the rotation angle and direction of the lower‐limb joints provides a feasible solution in such healthcare monitoring and rehabilitation applications. For instance, additional tests besides the common clinical assessment are generally required to elicit informative gait or balance features to further trim the differential diagnosis, in which lower‐limb motion monitoring in real time would be highly beneficial. To demonstrate the R‐TENG's capability in lower‐limb motion sensing to supplement future clinical diagnosis, we have attached an R‐TENG on the knee joint to measure three imitated gait features, i.e., slightly abnormal, highly abnormal, and reduced strength, as shown in **Figure** **5**a. In a two‐min test, the subject marches on the spot with a frequency of 0.5 Hz, during which the maximum knee bending angles per cycle are calculated through the pulsed triboelectric output and recorded. A more random pattern with larger variations on the knee rotation angle can be observed in the highly abnormal gait, while more regular yet smaller knee angles are detected with reduced muscle strength. In addition, the R‐TENG has also been demonstrated to monitor a typical Parkinsonian gait, where freezing of gait (FOG) is detected after a loss of stride period (Figure 5b). The small and irregular peaks imply the patient's temptation to move but are limited by the impaired mobility, which is common to PD patients when encountering certain events such as turning. Since FOG is now increasingly recognized as a major cause of falls in PD patients, the immediate detection of the sudden freeze would be helpful to prevent potential harm.

Apart from the directly measurable lower‐limb joint bending angles, step length is also a vital gait feature reflecting gait disorders to a certain extent, which are rarely measured directly for diagnosis. Assembling R‐TENGs on the sides of hip to measure the hip and thigh rotation angle in a gait cycle, we can calculate the step length of the lower limbs, as depicted in Figure 5c. Photos of R‐TENGs attached to hip joints on both legs with the corresponding angles under different walking states are shown in Figure S11 (Supporting Information). To be clarified, a step length is defined as the linear distance between two successive gait events of the same type on opposite limbs, e.g., left heel contact and right heel contact. Schematic showing the step duration of the left limb and right limb is provided in Figure 5c(iii) for better illustration. The detailed calculation process and adopted equation are provided in Figure S12a (Supporting Information). Here, the outputs of R‐TENGs on both limbs are measured with five‐step lengths from 35 to 75 cm (Figure 5c(ii)), with the corresponding angles between two thighs measured as shown in Figure S12b (Supporting Information). We compared the measured thigh angle from the R‐TENG's output, the calculated thigh angle based on the known step lengths, and the reference thigh angle obtained through photos taken from the side view, where the thigh angle is defined as the sum of the rotation angles from both limbs. Good consistency between the measured values and the reference values can be observed, showing a satisfactory accuracy of step length estimation with the R‐TENG. Compared to the visual observation widely adopted in current clinical assessment, the R‐TENG provides statistical measurement data regarding the important gait/posture features, such as the knee bending angle, hip rotation angle, and step length. In such a case, it would function as a supplementary platform to offer specific signs and leads to a more broadened differential diagnosis.

For the elderly or patients suffering impaired lower‐limb mobility, exercise and physical therapy are generally encouraged as efficacious adjuncts to medicines. However, there still lacks an efficient tool to track the strength and frequency of the performed exercises, especially considering they stay outside of the hospital most of the time. Therefore, it is hard for the physician to evaluate the effectiveness of the rehabilitation exercises and to track the improvement on the patient physical capacity after physical therapy in time. Taking the 2‐min step test as an example, the participants are instructed to march on the spot for two minutes, and the total number of times the right knee reaches the required height reflects the participant's aerobic capacity. Meanwhile, similar postures are also encouraged in some balance exercises to improve the gait/posture stability of the elderly.^[^ ^117^, ^118^, ^119^ ^]^ With the sensory information from the R‐TENG on the hip, we are able to estimate the heights of the foot from 5 to 40 cm, as shown in Figure 5d. The corresponding postures and the detailed calculation process are provided in Figures S13 and S14a (Supporting Information), with the comparison of calculated and reference angles in Figure S14b (Supporting Information). Good consistency between the calculated height and reference height can be observed, implying the adequate accuracy of this methodology for foot height estimation. This additional information apart from the joint rotation angle would be beneficial for task‐specific monitoring in clinical assessment and rehabilitation applications. A video showing the R‐TENG assisting the quantitative measurement in a dynamic balance training is provided as Video S1 (Supporting Information). In this demonstration, the subject imitates the possible postures of a patient before and after rehabilitation exercises. The improvement of the subject's physical capacity and gait/posture stability can be quantitatively measured. Such information would be highly significant for both the patient and the physician, enabling adequate supervision of the home‐based training and opportune evaluation of the patient's physical capacity.

## 6 Sports Monitoring and VR Application

Considering the capability of the R‐TENG for lower‐limb motion monitoring, its applications can be further expanded from rehabilitation monitoring to HMI, e.g., complex control in VR space, which is appropriate to assist coaches in physical training or skill training. Based on the quantitative detection of the multimotion for the lower‐limb joints, an R‐TENG‐based VR interface is developed to perform more variously complicated activities with proper consistency and effectiveness. Furthermore, the signal acquisition system operated by the sensor–MCU frame can greatly achieve the virtual interaction between real space and cyberspace without privacy infringement.

**Figure 6:**
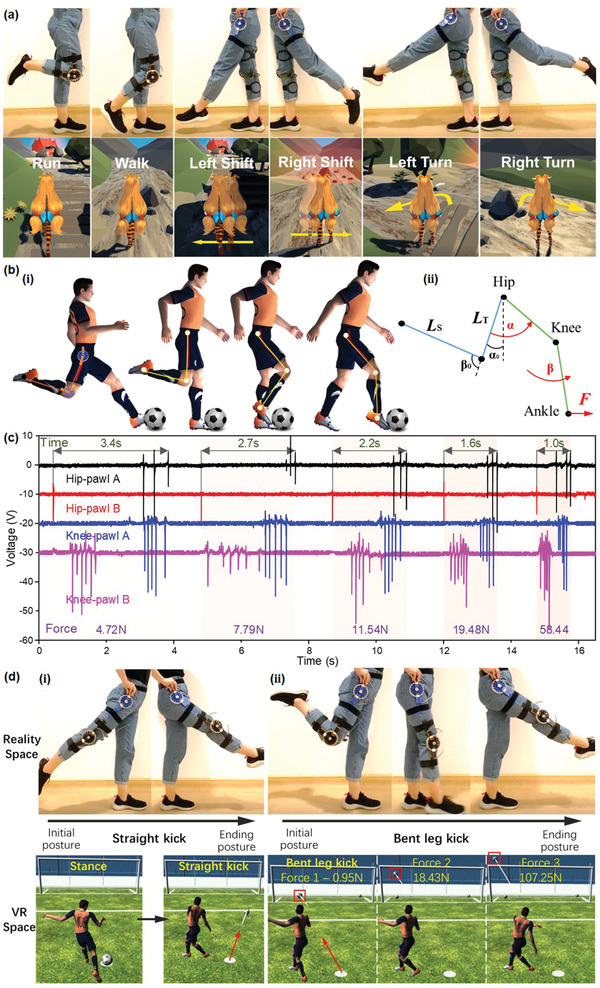
Human‐machine interfacing in VR and sports monitoring applications. a) Demonstration of VR control game with R‐TENGs distributing on the left hip and right knee joints to trigger multiple instructions of moving directions and multiple motions, respectively. b) Detailed motion segments of a kicking process with two R‐TENGs fixed on the hip and knee joints (i) and the schematic diagram of initial and ending postures (ii). c) Signal patterns of five football kicking motions with ascending forces. d) Demonstration of football kicking VR control with two kinds of kicking motions: (i) straight kick, (ii) bent leg kick under different kicking forces.

To explore the functions as a basic HMI in VR games, here we designed a lower‐limb‐based treasure hunting game with R‐TENG acting as the hardware to realize the control of different motions and specific positions. As shown in **Figure** **6**a, the R‐TENGs are distributed on the left hip and right knee joints to trigger the instructions of moving directions and multiple motions, respectively. There are six actions being demonstrated including two motions (walk and run) and four direction‐based motions (right/left shift and right/left turn). The bending movement of the right knee is defined as commands to control the virtual figure to walk or run regarding the bending angles. To initiate running, a bending degree reaching the preset threshold value (60°) needs to be generated, resulting in four peaks in both channels representing a complete bending cycle (Figure S15a, Supporting Information). Similarly, the walking motion can be activated by a minor bending degree, such as 15°, as shown in Figure S15b (Supporting Information). For the direction‐based motions, the right hip sensor experiencing the complete kicking forward or backward triggers the left shift/turn or right shift/turn, in which case the rotation degree determines the shift or turn motion (Figure S15c‐f, Supporting Information). A detailed video showing a participant playing this treasure‐hunting game with the R‐TENGs can be found in Video S2 (Supporting Information). It should be noted that the control scheme is not limited to current combinations, i.e., the users can define their own commands with the R‐TENGs attached to lower‐limb joints wherever they feel intuitive and convenient for themselves. Accordingly, the incorporation of hip and knee sensors with multiple channel outputs can fulfill multimotion to mimic different moving statuses in virtual space, and hence, this approach plays a key role in creating and flourishing game varieties to enrich the enjoyment and experience.

Targeting specific application scenarios such as sports training, the R‐TENG located on the body can collect a range of biomechanical data designed to improve performance, recovery, or other aspects of health. Accordingly, a demonstration of football kicking monitoring is presented, which is designed to be a training program for monitoring the lower‐limb trajectory and speed with a further calculation of kicking force during a specific strike. The kicking process detailing to few motion segments is shown in Figure 6b(i) with two R‐TENGs fixed on the hip and knee joints. The schematic diagram of initial and ending postures is illustrated in Figure 6b(i). Generally, the pulsed output from different channels implies the rotation angle of the knee or hip, which reflects the entire kicking trajectory with the rotation angles measured as illustrated in Figure 6b(ii). The four‐channel output of five kicking motions with ascending forces from 4.72 to 58.44 N is sketched in Figure 6c. With the detected angle values, we can estimate the kicking force as demonstrated in Note S1 (Supporting Information), with a comparison between measured and calculated values plotted in Figure S16 (Supporting Information). For example, the kicking force along with a particular angular velocity for a lasting period of 2.7 s is derived as 8.84 N, and the actual effective force tested by kicking the force meter is 7.79 N. This kinetic analysis not only can track the motion accuracy but also offer an effective method to quantify the proper force to fulfill a scientific training and testing analyzed by coaches. As a practical demonstration based on the previous analysis, the football training program is designed and performed with the R‐TENGs (see Video S3 in the Supporting Information). As shown in Figure 6d, two kinds of kicking motions are conducted including a straight kick (Figure 6d(i)) and a bent leg kick (Figure 6d(ii)). Once the initial postures of the kicking motions are performed, a corresponding stance status will be ready in VR space. In this training program, the straight kick is set as the command for kicking the football to the right side, while the bent leg kick sets off the football motions to the left area. Based on the aforementioned kicking force calculation process, we can control the distance of the football trajectory in the VR space regarding the actual kicking strength. As shown in Figure 6d(ii), a stronger kick with a higher speed will trigger the football to move further as highlighted by the red frames.

In general, the proper utilization of R‐TENG placed on the lower‐limb joints can effectively explore the capabilities of performing multifunctional monitoring toward sports monitoring and complex VR applications. On the one hand, this sensing system provides an economical and efficient solution for capturing complex human motions. On the other hand, with the further specific analysis and calculation of the acquired original data, the system introduces a facile strategy of expanding its functionalities to estimate more kinematic quantities of interest.

## 7 Conclusion

In summary, an MC–EH–HL system is developed through the integration of a sliding block–rail‐based piezoelectric bimorph array generator and R‐TENG. The S‐PEG is specifically designed to leverage kinetic energy from lower‐limb activity in a way that the 3D motion trace is converted into 1D linear sliding on the rail, which activates the vibration of multiple piezoelectric bimorphs for energy harvesting. The interconnected sliding blocks of the S‐PEG enable the deflection of the PZT bimorphs whenever there is a rotation motion of the knee joint, which also allows for the integration of overall 20 bimorphs on one limb to boost up the energy converting performance. Owing to this unique structure, high power of 2.4 mW has been successfully achieved under a very low operation frequency (0.75 Hz). A high charging speed of 160 µC s^−1^ on a 1 mF capacitor can be achieved in the meantime. Combining the power management circuit, energy storage unit, MCU, and wireless transmission module, self‐sustainable IoT systems based on the S‐PEG are then demonstrated. The large output power of the S‐PEG enables a self‐sustained IoT system with wireless temperature and humidity sensing capabilities operated continuously. To track the lower‐limb postures, a bidirectional triboelectric rotation sensor (R‐TENG) is designed based on the delicate ratchet mechanism, which can be attached to multiple lower‐limb joints for real‐time monitoring. This distinctive mechanical structure contributes to a digitalized output pattern, where the rotation angle can be simply detected by the pulse numbers with minimal interference from environmental noises. Meanwhile, the structure design offers excellent tunability of the size and resolution by altering the dimensions of the mechanical pattern with improved fabrication procedures. On top of that, extra physical parameters related to the lower‐limb motions have also been estimated through kinetic analysis, such as raised foot height, step length, kicking force. Finally, such sensory information is adopted to demonstrate the R‐TENG's prospect in multiple application scenarios including rehabilitation monitoring, HMI for VR games, and sports monitoring. Looking forward, the MC–EH–HL system has offered a cost‐effective and energy‐saving solution for lower‐limb motion monitoring bypassing the inertial sensor, force sensor, and cameras, which exhibits great potential in future home‐based rehabilitation, VR gaming, and sports coaching applications.

## 8 Experimental Section

### Fabrication of the S‐PEG and R‐TENG

The components of the frame, base, and the ratchet wheel, pawl, and inhibiting components of R‐TENG was fabricated separately by the 3D printer (ANYCUBIC 3D‐Printer 4Max Pro) using polylactic acid filament, which all were designed by SolidWorks 2019. The connecting rod of S‐PEG was selected as aluminum alloy and fabricated by laser cutting.

### Assembly of the S‐PEG

The connection between frame and base, PZT bimorphs (http://www.pantpiezo.com) inserted into base were glued tightly. The fixation of the bearing (686ZZ: 6 mm inner diameter, 13 mm external diameter, and 5 mm height) with the sliding block and connecting rod was interference fit, which resulted in the restriction in the axial direction of bearing but free in the rotating direction. Similarly, the sliding rail configured in the frame was limited between the left and right sides. Besides, there were four hollow‐carved designs on the bottom of each frame prepared for equipping the Velcro tape to attach to the legs for human body wearing.

### Assembly of the R‐TENG

To complete the circuition process of the ratchet wheel and pawls, the wheel and two inhibiting components shared a stationary shaft by using three same bearings (686ZZ), which permitted each component to separately rotate smoothly. The inhibiting components consisted of three parts: an L‐shaped structure with circular hollows, respectively, located at the long handle edge and the center of the short horizontal rectangle, a short L‐shaped structure with small insertion protrusion and a hollow same as above, and a stick shaft across the center of two L‐shaped structures. To limit the side position of the pawl trapped in the inhibiting components, two small rectangle acrylic boards along with the L‐shaped structures were strapped together by tape. The triboelectric positive material of PET adhered to an Al electrode wrapped around the pawl to transfer the charges neatly without affecting the circulate sliding. In order to prevent the wires when attached to the teeth from getting tangled as they rotated, the negative triboelectric layer here was first pasting the lengthened Al electrodes with longer side sticking to the side face of wheel teeth and then adhering to the PTFE above. With 24 extending electrodes on the side face, a circinate copper (Cu) electrode connected all Al electrodes and another two Cu electrodes wrapped on the L‐structures to realize a completely unaffected rotation and charges transfer of the wheel.

### Test Setup

The piezoelectric open‐circuit voltage, transferred charges, and short‐circuit current were measured by an electrometer (Model 6514, Keithley). The signals were displayed and recorded by an oscilloscope (Agilent, InfiniVision, DSO‐X 3034A) with a high voltage probe (Keysight N2771B, 100 MΩ) being prepared for the triboelectric sensor measurement. The Bluetooth module was made from commercial Bluetooth low energy (BLE) sensors (CYALKIT‐E02), with integrated temperature and humidity sensors (Si7020‐A20). The demonstration of the VR scenario was controlled by the Python code, which was delivered from the triboelectric signals processed by Arduino MEGA 2560 and a conditioner‐printed circuit board.

## Conflict of Interest

The authors declare no conflict of interest.

## Supporting information

Supporting Information

Supplemental Video 1

Supplemental Video 2

Supplemental Video 3

## Data Availability

The data that support the findings of this study are available from the corresponding author upon reasonable request.
