# Supplementary material for: A Motion Capturing and Energy Harvesting Hybridized Lower‐Limb System for Rehabilitation and Sports Applications
Source: Adv Sci (Weinh). 2021 Aug 19;8(20):2101834. doi: 10.1002/advs.202101834 (PMC8529439; doi:10.1002/advs.202101834)
Supplement: Supplementary file 1 — Supporting Information [file ADVS-8-2101834-s001.pdf]

WILEY-VCH

© 2021 Wiley-VCH GmbH

ADVANCED SCIENCE
Open Access

Supporting Information

for *Adv. Sci.*, DOI: 10.1002/advs.202101834

A motion capturing and energy harvesting hybridized lower-limb system for rehabilitation and sports applications

Shan Gao, Tianyiyi He, Zixuan Zhang, Hongrui Ao, Hongyuan Jiang, and Chengkuo Lee**

Supporting Information

A motion capturing and energy harvesting hybridized lower-limb system for rehabilitation and sports applications

Shan Gao, Tianyiyi He, Zixuan Zhang, Hongrui Ao, Hongyuan Jiang, and Chengkuo Lee**

Table S1. The basic design criteria of the ratchet wheel.
Table S2. Charging rate comparison with the reported energy harvesters. Comparison of the charging rate between previously proposed energy harvesters of different mechanisms and the proposed S-PEG in this work.

Figure S1. The detailed dimensions of the (a) R-TENG, (b-i) S-PEG, and (b-ii) PZT bimorph and sliding block-rail. And the total volume and mass of S-PEG are 109.073g and 142.341g, respectively.
Figure S2. The working process of the R-TENG. (a) The initial state of the ratchet wheel and two pawls, where the red star represents the labeled tooth. When pawl B is fixed, (b) pawl A is rotating in the direction of wheel teeth while the ratchet wheel is fixed; (b) pawl A is rotating back in the counter-direction of wheel teeth while the ratchet wheel is rotating together, as shown in the position of the labeled tooth.
Figure S3. (a) The schematic diagram of interference length Δ between bimorph and cylinder, which is enlarged at the red circle. (b) The output voltage under different amplitudes of the PZT bimorph controllable by changing the length of the interference.
Figure S4. (a) The schematic diagram of the connection rod length and wearing position on the lower limb, (b) photos of S-PEG on right lower limb of the user, and the calculative process.
Figure S5. The output of S-PEGs under the connection in series. (a) the rectified output voltages of ten individual bimorphs, (b-c) the output current and voltage of the parallel-connected PZT bimorphs, (d) charging curves of ten PZT bimorphs in series and parallel to charge a 47 μF capacitor through conventional bridge rectifiers.
Figure S6. Output voltages of 90 degrees rotary angles under different environment humidity ranging from 30% to 90%.
Figure S7. (a) The short-circuit current signal with corresponding angles (0-90°) and R-TENG screenshot, (b) the knee bending angles (0-90°) equipped on the knee.
Figure S8. The corresponding screenshot of different lower-limb actions: (a) Thigh backward kicking, (b) thigh forward kicking with R-TENG on the hip, (c) shank backward kicking with R-TENGs on the knee.
Figure S9. The electronic components setup of the MC-EH-HL system with the detailed channels and ports connection.

Figure S10. The humidity and temperature sensor is powered by S-PEGs with the assistance of a power management chip of LTC-3588-1. (a) The schematic diagram of circuit design, (b) the photo of the testing setup.

Figure S11. The corresponding screenshot of different step lengths (in the middle of the figure) and angles (at the bottom of the figure) between right and left thigh with two R-TENGs on each hip. (a-b) Right view exhibits the angle between the right leg and vertical axis and the length between left tiptoe and right heel, (c-d) Left view exhibits the angle between the left leg and vertical axis.

Figure S12. (a) The schematic diagram of the lower-limb walking model, where L_T, L_S are the length of thigh and knee, α_1, α_2 demonstrate the angle of right and left thigh, respectively and $\alpha_M=\alpha_1+\alpha_2$, s is the step length and $s=(L_T+L_S)(\sin\alpha_1+\sin\alpha_2)$. (b) The comparison among the measured angles, calculated values, and reference data under different walking step lengths. The differences between these results demonstrate an average deviation of about 11.684%, which emerges a better consistent performance in the middle step length than extreme distances.

Figure S13. The corresponding screenshot of different heights (5-40cm) and angles (30-90°) with the R-TENG on the right hip.

Figure S14. (a) The schematic diagram of the lower-limb high-knee model, where L_T, L_S are the length of thigh and knee, α demonstrates the angle between right and left thigh and h is the height of the knee lifting, where $h=L_T(1-\cos\alpha)$. (b) The measured angles present less than 3.633% of average deviation with the reference and calculated data, which demonstrates the accuracy output of the R-TENG.

Figure S15. The output voltage of VR games with the R-TENGs on the left hip and right knee joint under different motions (a) Running, (b) Walking, (c) Left shifting, (d) Right shifting, (e) Right turning, (f) Left turning.

Figure S16. The comparison between the measured forces and calculated results under different speeds of football kicking motion on the hip and knee joints.

Note S1. Lower-limb model of the thigh and shank segments under football tracking motion.

Video S1. Lower-limb monitoring in dynamic balance training with the R-TENG on the knee joint.

Video S2. A treasure-hunting VR game with the R-TENGs on the hip and knee joints.

Video S3. A football training program with the R-TENGs on the hip and knee joints.

Table S1. The basic design criteria of the ratchet wheel.

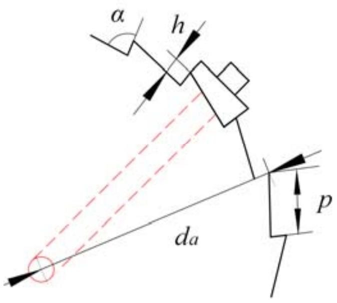

Parameter	Formula	Values
Teeth *z*	8~250	24
Module *m*	1, 1.5, 2, 2.5, 3, 3.5, 4, 5, 6, 8, 10	2.5
Diameter *da*	*da*=*mz*	100mm
Space Width *p*	*p*=π*m*	7.854mm
Height of Tooth *h*	*h*=0.75*m*	1.875mm
Tooth Space Angle *α*	60~80°	80°
Width of Rachet *b*	*b*=(1~4)*m*	10mm

Teeth *z*: Edge of the ratchet, consisting of uniform but asymmetrical shapes. Each tooth has a moderate slope on one edge and a much steeper slope on the other edge.

Module *m*: Module of the ratchet. It is a scaling factor that defined by the mechanical engineers as $m=p/\pi$, where *p* is the circular pitch.

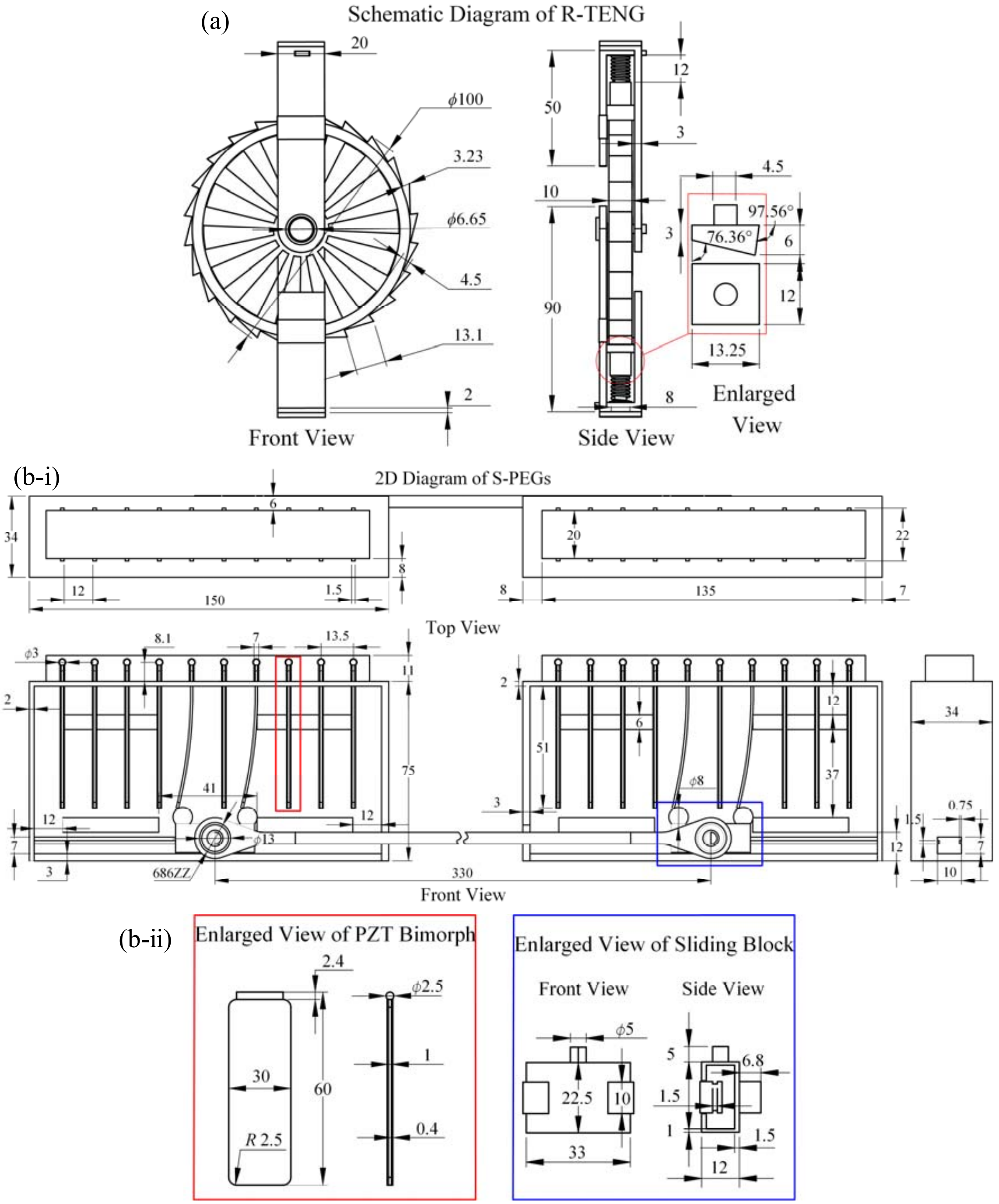

Figure S1. The detailed dimensions of the (a) R-TENG, (b-i) S-PEG, and (b-ii) PZT bimorph and sliding block-rail. And the total volume and mass of S-PEG are 109.073g and 142.341g, respectively.

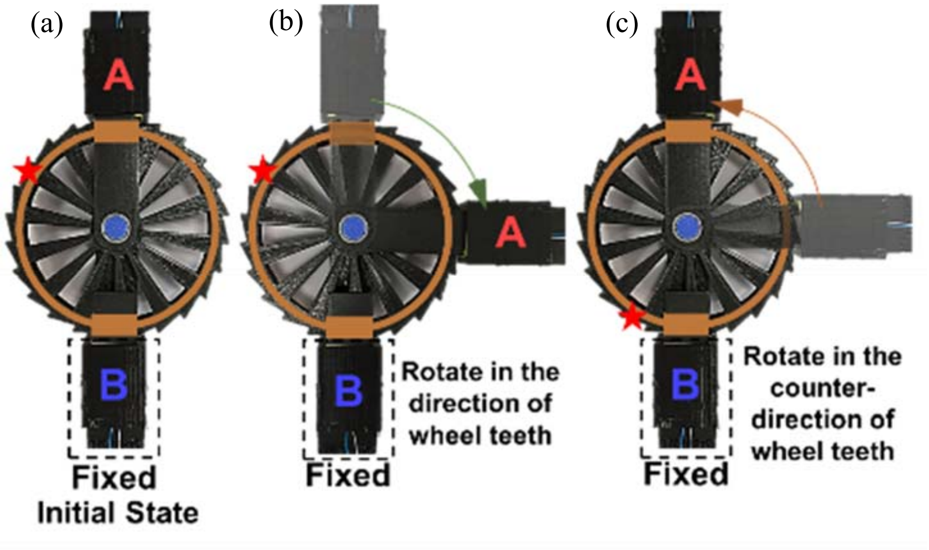

Figure S2. The working process of the R-TENG. (a) The initial state of the ratchet wheel and two pawls, where the red star represents the labeled tooth. When pawl B is fixed, (b) pawl A is rotating in the direction of wheel teeth while the ratchet wheel is fixed; (b) pawl A is rotating back in the counter-direction of wheel teeth while the ratchet wheel is rotating together, as shown in the position of the labeled tooth.

(a)

(b)

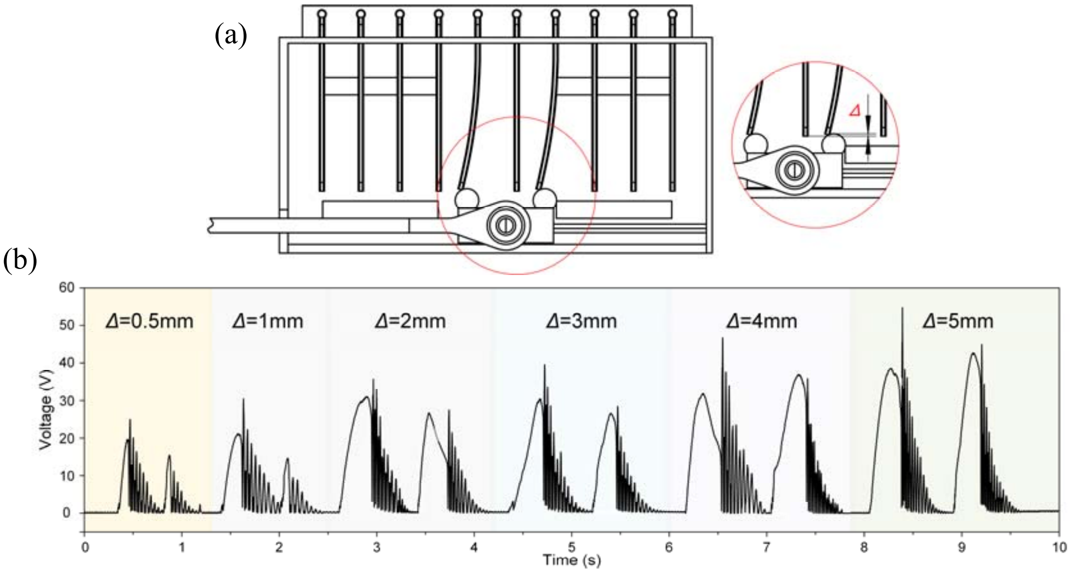

Figure S3. (a) The schematic diagram of overlapping area Δ between bimorph and cylinder, which is enlarged at the red circle. (b) The output voltage under different amplitudes of the PZT bimorph controllable by changing the overlapping area.

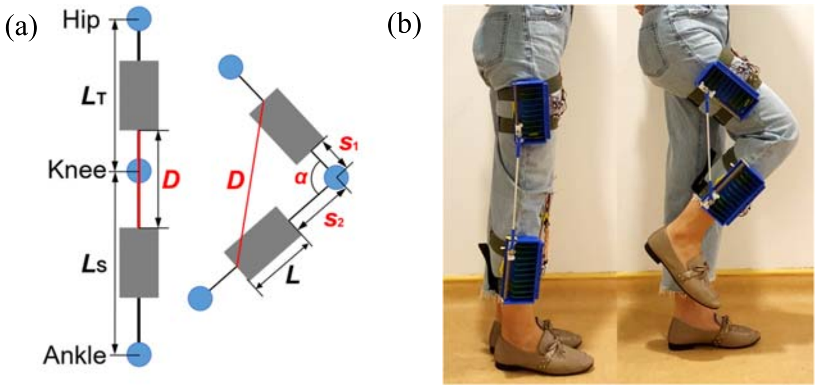

Figure S4. (a) The schematic diagram of the connection rod length and wearing position on the lower limb, and (b) photos of S-PEG on right lower limb of the user. The calculative process is listed as below, where L_T=445mm, L_S=475mm of the user, and L=150mm, 45°≤α<180°. The relationship between D, s_1 and s_2 is:

$$\begin{cases} D = s_1 + s_2 \\ D^2 = (s_1 + L)^2 + (s_2 + L)^2 - 2(s_1 + L)(s_2 + L)\cos\alpha \end{cases}$$

where $\begin{cases} 100\text{mm} \le s_1 < L_T - \text{L} \\ 100\text{mm} \le s_2 < L_S - \text{L} \end{cases}$.

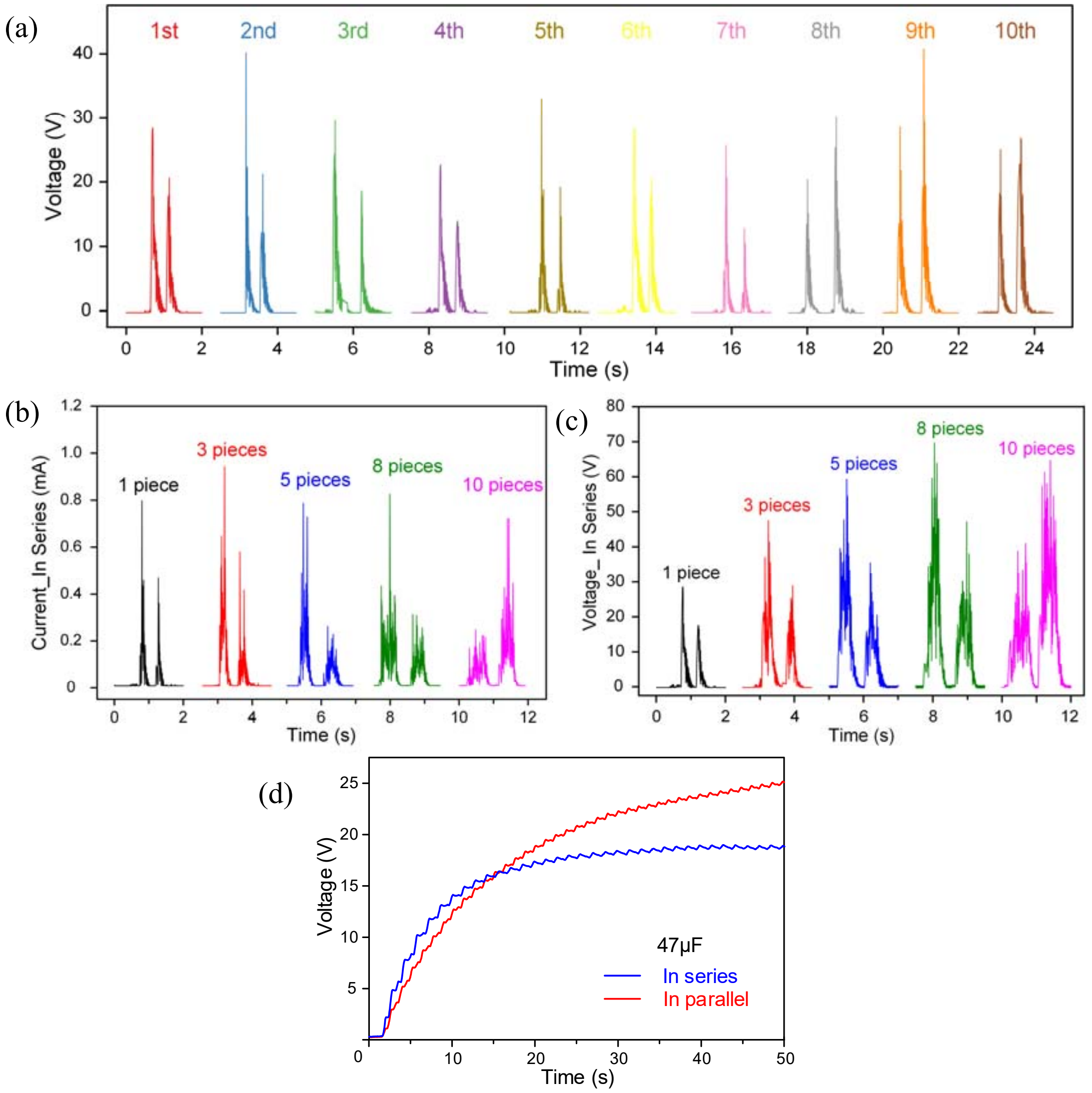

Figure S5. The output of S-PEG under the connection in series. (a) the rectified output voltages of ten individual bimorphs, (b-c) the output current and voltage of the parallel-connected PZT bimorphs, (d) charging curves of ten PZT bimorphs in series and parallel to charge a 47 µF capacitor through conventional bridge rectifiers.

Table S2. Charging rate comparison with the reported energy harvesters of different mechanisms.

Ref.	Year	Mechanism	Charge Speed [μC/s] [a)]	Motion speed or frequency
[60]	2020	Triboelectric	1	1Hz
[71]	2019	Triboelectric	2.47	1Hz
[86]	2021	Triboelectic	2.625	5Hz
[95]	2019	Triboelectric	3.178	2Hz
[53]	2018	Piezoelectric	4.125	1.2Hz
[73]	2021	Piezoelectric	8.356	4Hz
[92]	2021	Piezoelectric	103.13	25Hz
Our work	2021	Piezoelectric	160	0.75Hz

[a)] The charging speed per volume is estimated by $\Delta Q/t = C \times U/t$, where the accumulated charge (ΔQ) equals to the product of capacitor size (C) and the charged voltage (U).

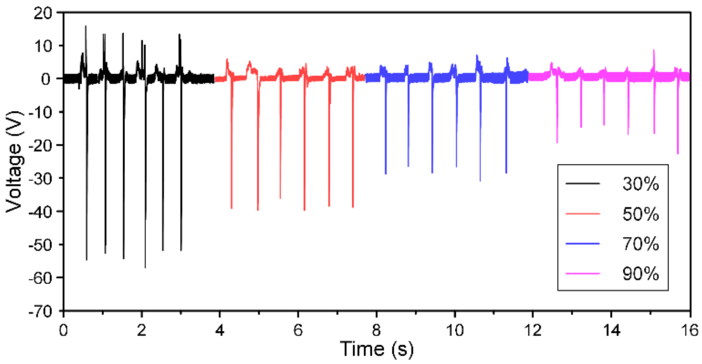

Figure S6. Output voltages of 90 degrees rotary angles under different environment humidity ranging from 30% to 90%.

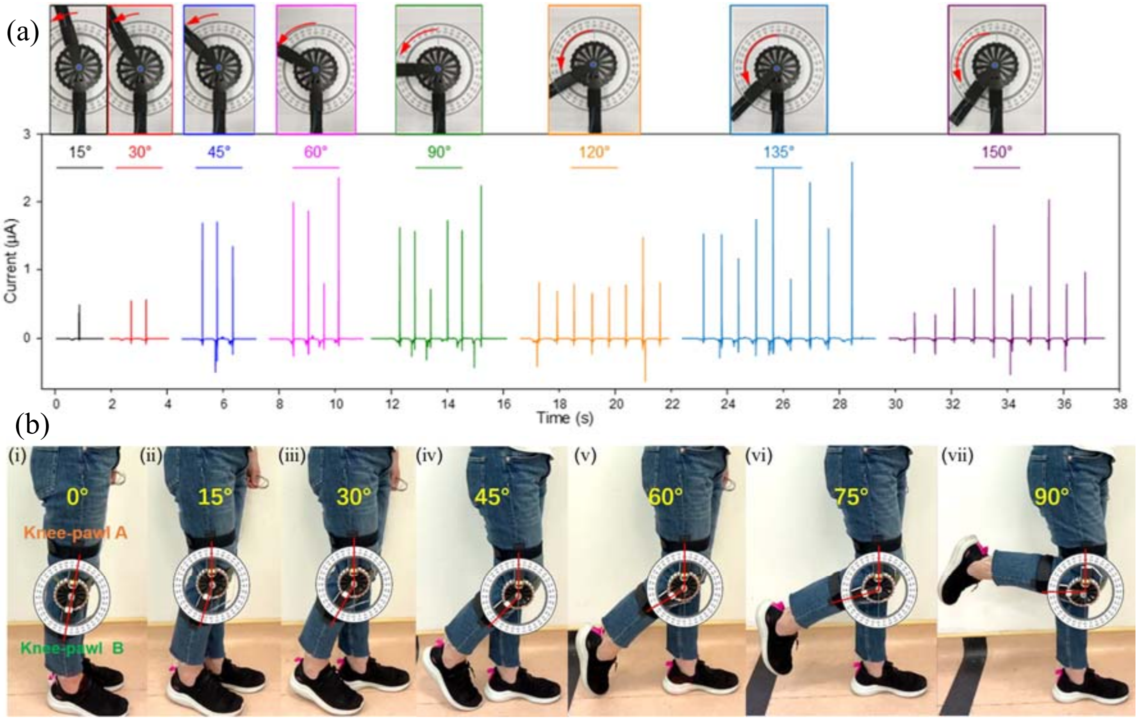

Figure S7. (a) The short-circuit current signal with corresponding angles (0-90°) and R-TENG screenshot, (b) the knee bending angles (0-90°) equipped on the knee.

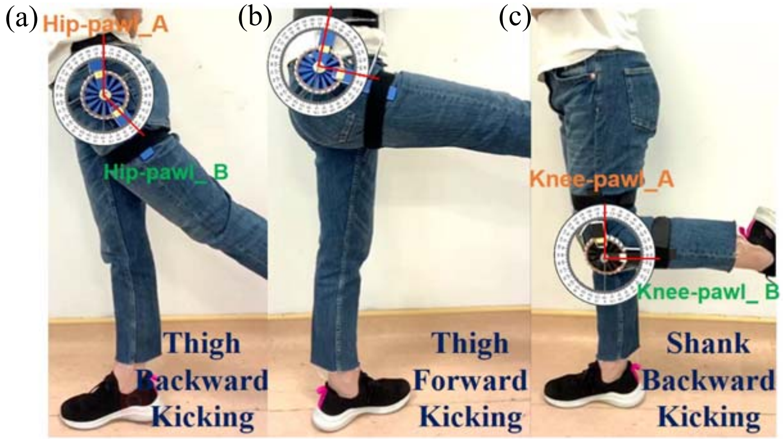

Figure S8. The corresponding screenshot of different lower-limb actions: (a) Thigh backward kicking, (b) thigh forward kicking with R-TENG on the hip, (c) shank backward kicking with R-TENGs on the knee.

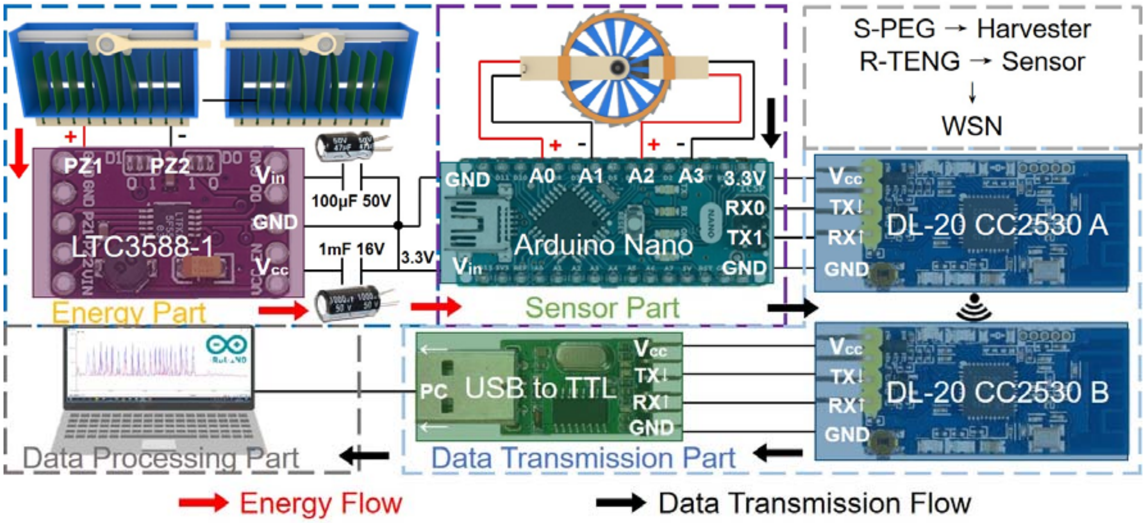

Figure S9. The electronic components setup of the MC-EH-HL system with the detailed channels and ports connection.

(a)

(b)

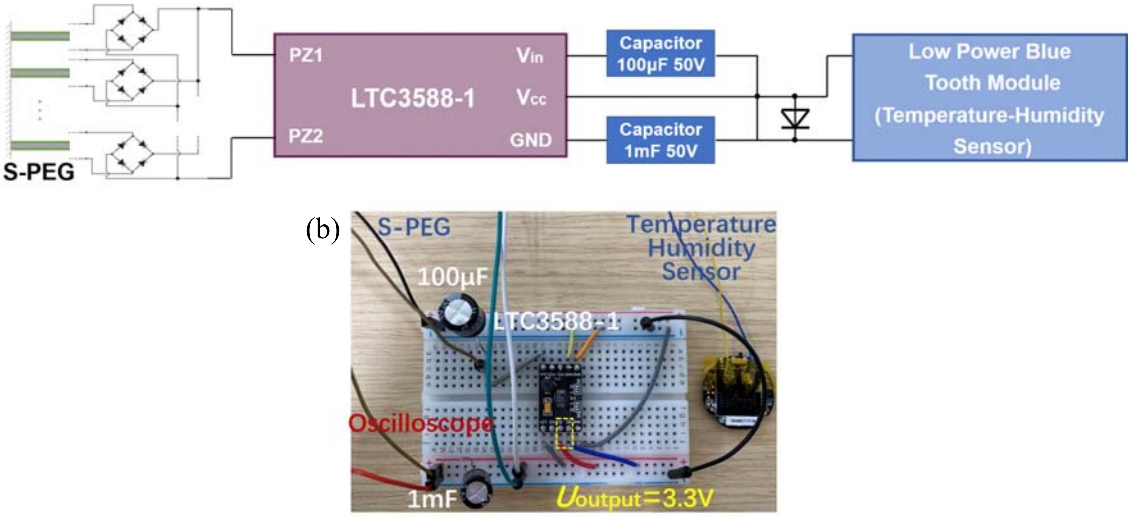

Figure S10. The humidity and temperature sensor is powered by S-PEG with the assistance of a power management chip of LTC3588-1. (a) The schematic diagram of circuit design, (b) the photo of the testing setup.

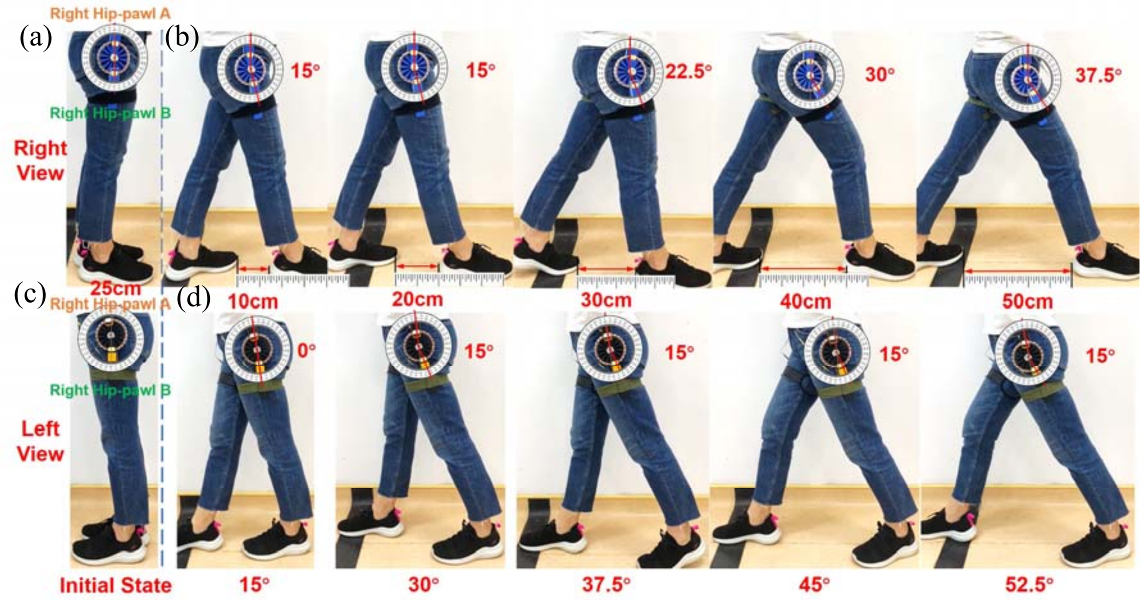

Figure S11. The corresponding screenshot of different step lengths (in the middle of the figure) and angles (at the bottom of the figure) between right and left thigh with two R-TENGs on each hip. (a-b) Right view exhibits the angle between the right leg and vertical axis and the length between left tiptoe and right heel, (c-d) Left view exhibits the angle between the left leg and vertical axis.

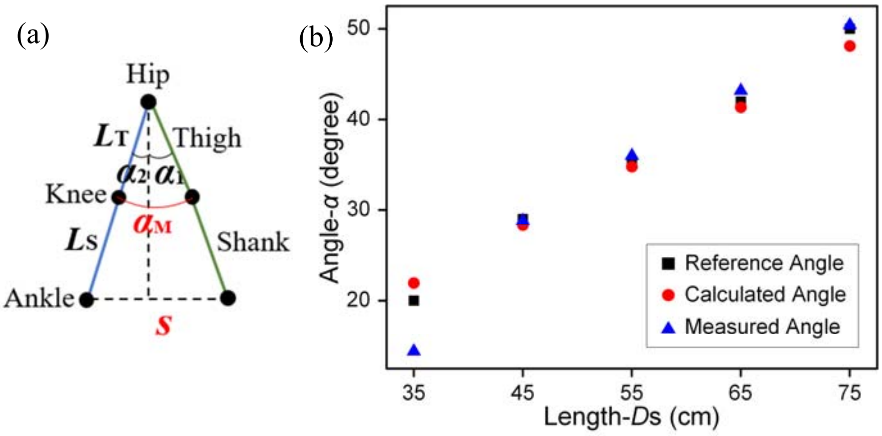

Figure S12. (a) The schematic diagram of the lower-limb walking model, where L_T, L_S are the length of thigh and knee, α_1, α_2 demonstrate the angle of right and left thigh, respectively and $\alpha_M=\alpha_1+\alpha_2$, s is the step length and $s=(L_T+L_S)(\sin\alpha_1+\sin\alpha_2)$. (b) The comparison among the measured angles, calculated values, and reference data under different walking step lengths. The differences between these results demonstrate an average deviation of about 11.684%, which emerges a better consistent performance in the middle step length than extreme distances.

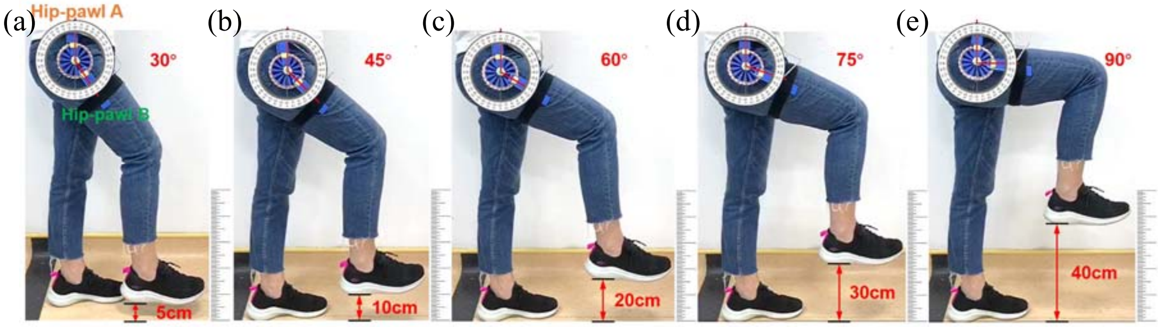

Figure S13. The corresponding screenshot of different heights (5-40cm) and angles (30-90°) with the R-TENG on the right hip.

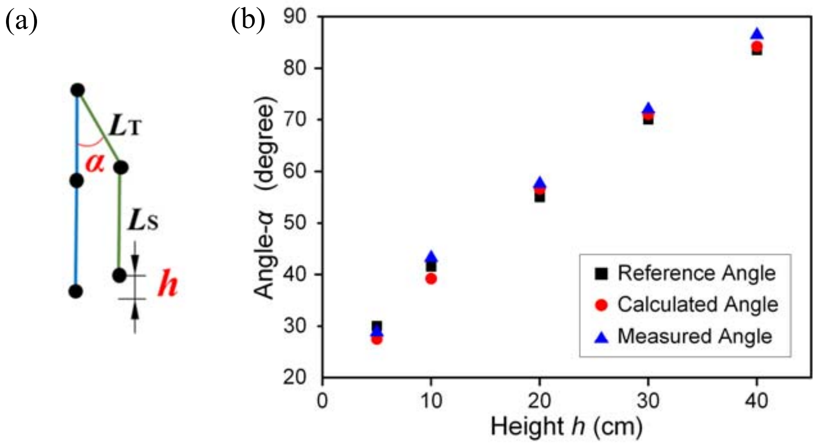

Figure S14. (a) The schematic diagram of the lower-limb high-knee model, where L_T, L_S are the length of thigh and knee, α demonstrates the angle between right and left thigh and h is the height of the knee lifting, where $h = L_T(1 - \cos\alpha)$. (b) The measured angles present less than 3.633% of average deviation with the reference and calculated data, which demonstrates the accuracy output of the R-TENG.

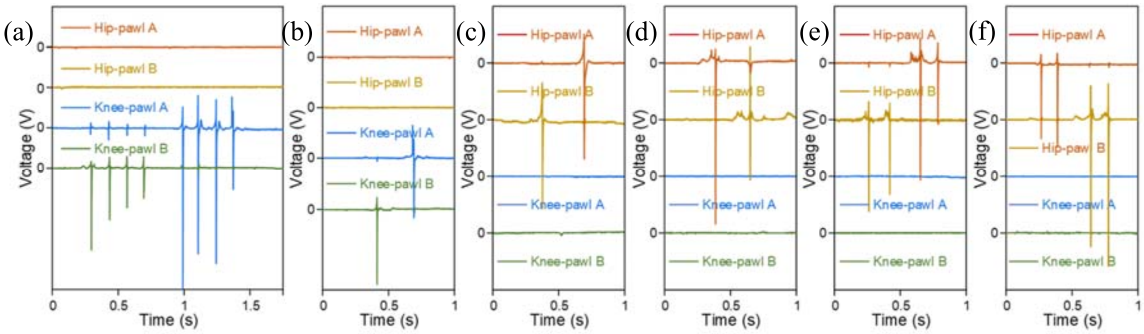

Figure S15. The output voltage of VR games with the R-TENGs on the left hip and right knee joint under different motions (a) Running, (b) Walking, (c) Left shifting, (d) Right shifting, (e) Right turning, (f) Left turning.

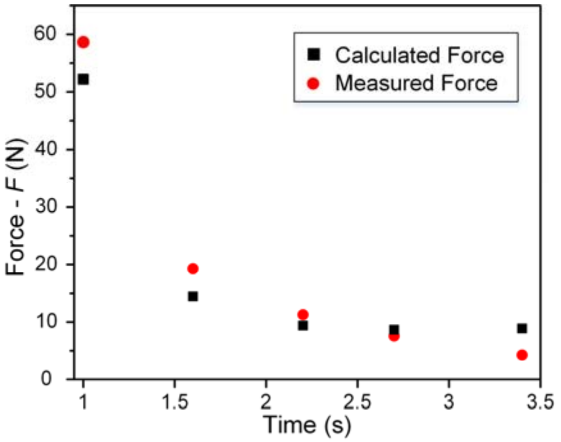

Figure S16. The comparison between the measured forces and calculated results under different speeds of football kicking motion on the hip and knee joints.

Note S1. Lower-limb model of the thigh and shank segments under football tracking motion.

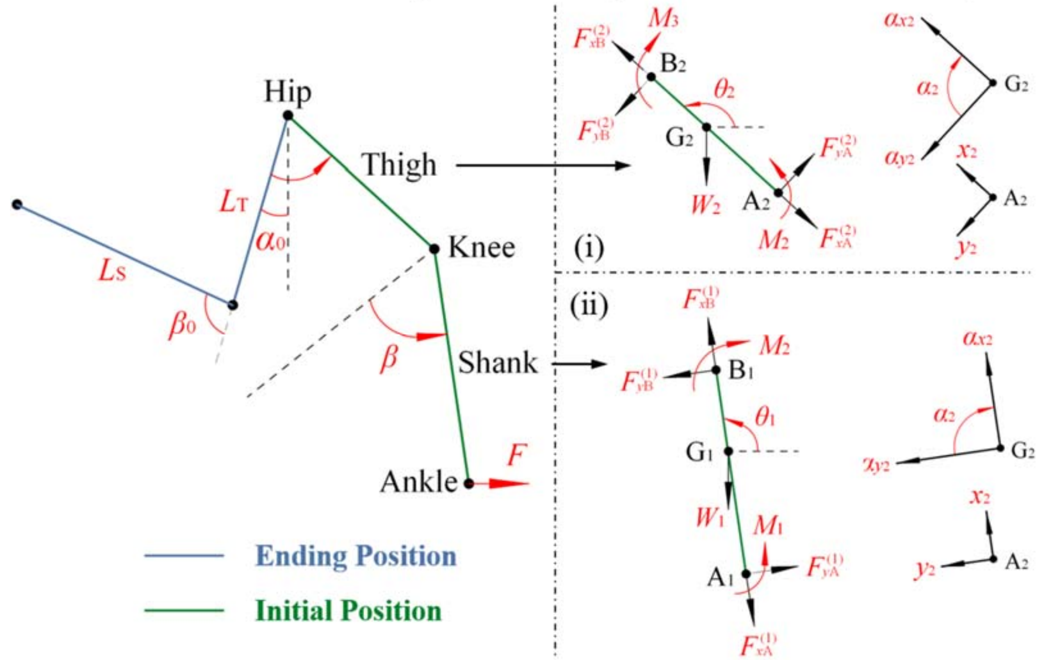

Nomenclature:

1) Subscripts:

T (2): thigh.

S (1): shank.

3: ankle.

K: knee.

G: mass center.

x, *y*: coordinates of the lower limb.

A, B: top and bottom position of the segment.

2) Variable in equations:

g: gravitational constant (9.81m·s^{-2}).

m: segment mass.

R_T, R_K, R_S: radius of base of the thigh, knee, and mid-shin.

L: segment length.

L': distances between a segment's proximal joint and mass center.

α_0, β_0: initial position of the thigh and shank angles.

α, β: angles of thigh and shank between initial and end positions.

θ: segment orientation angle measured in a counterclockwise direction from a horizontal line at the proximal joint to the longitudinal axis of the segment.

Φ: relative angle of lower limb.

W: weight.

I: moment of inertia.

ω: angular velocity.

α_1, α_2: angular accelerations of thigh and shank.

a: linear acceleration.

M: torque.

F: force.

Football kicking diagram for the thigh and shank segments are shown in the figure. Inertial system to the coordinate system transformation is used for convenience in the equations of motion for each segment. From the free-body diagrams demonstrated in (i) and (ii), the following equations of motion are formulated:[1]

(i) Shank:

$$\begin{cases} F_{x\mathrm{B}}^{(1)} - F_{x\mathrm{A}}^{(1)} - W_1 \sin\theta_1 = m_1 a_{x1} \\ F_{y\mathrm{B}}^{(1)} - F_{y\mathrm{A}}^{(1)} - W_1 \cos\theta_1 = m_1 a_{y1} \end{cases},$$

$$M_2 - M_1 + F_{y\mathrm{A}}^{(1)}\left(L_\mathrm{S} - L'_\mathrm{S}\right) + F_{y\mathrm{B}}^{(1)} L'_\mathrm{S} = I_1 \alpha_1 ,$$

where $L'_\mathrm{S} = \mathrm{G_1B_1}$.

(ii) Thigh:

$$\begin{cases} F_{x\mathrm{B}}^{(2)} - F_{x\mathrm{A}}^{(2)} - W_2 \sin\theta_2 = m_2 a_{x2} \\ F_{y\mathrm{B}}^{(2)} - F_{y\mathrm{A}}^{(2)} - W_2 \cos\theta_2 = m_2 a_{y2} \end{cases},$$

$$M_3 - M_2 + F_{y\mathrm{A}}^{(2)}\left(L_\mathrm{T} - L'_\mathrm{T}\right) + F_{y\mathrm{B}}^{(2)} L'_\mathrm{T} = I_2 \alpha_2 ,$$

where $L'_{\mathrm{T}} = \mathrm{G}_2\mathrm{B}_2$.

The continuity conditions at knee joint are as follows:

(iii) Knee:

$$\begin{cases} F_{x\mathrm{A}}^{(2)} = F_{x\mathrm{B}}^{(1)}\cos\phi_1 + F_{y\mathrm{B}}^{(1)}\sin\theta_1 \\ F_{y\mathrm{A}}^{(2)} = -F_{x\mathrm{B}}^{(1)}\sin\theta_1 + F_{y\mathrm{B}}^{(1)}\cos\theta_1 \end{cases},$$

where $\phi_1 = \theta_2 - \theta_1$.

The variables in the above equations can be calculated as:

$$\begin{cases} \theta_1 = 90° - \beta_0 + \beta + \dfrac{\alpha}{2} \\ \theta_2 = 90° + \alpha_0 - \alpha \end{cases}, \quad \begin{cases} I_1 = \dfrac{m_{\mathrm{S}}R_{\mathrm{K}}^2}{4} + \dfrac{m_{\mathrm{S}}L_{\mathrm{S}}^2}{12} \\ I_2 = \dfrac{3m_{\mathrm{T}}}{10}\cdot\dfrac{R_{\mathrm{T}}^4 + R_{\mathrm{T}}^3R_{\mathrm{S}} + R_{\mathrm{T}}^2R_{\mathrm{S}}^2 + R_{\mathrm{T}}R_{\mathrm{S}}^3 + R_{\mathrm{S}}^4}{R_{\mathrm{T}}^2 + R_{\mathrm{T}}R_{\mathrm{S}} + R_{\mathrm{S}}^2} \end{cases}.$$

According to the law of conservation of momentum, each segment can derive equations as:

$$\begin{cases} mg\Delta h = 0.5mv^2 \\ \Delta h = L(1-\cos\dfrac{\alpha}{2}) \\ v = L\omega \end{cases}, \text{ which can be wrote as } \omega = \sqrt{\frac{2g\left(1-\cos\dfrac{\alpha}{2}\right)}{L}},$$

that is,

$$\begin{cases} \omega_1 = \sqrt{\dfrac{2\mathrm{g}\left(1-\cos\beta/2\right)}{L_{\mathrm{S}}}} \\ \omega_2 = \sqrt{\dfrac{2\mathrm{g}\left(1-\cos\alpha/2\right)}{L_{\mathrm{T}}}} \end{cases}.$$

Therefore, other parameters can be calculated as:

$$\begin{cases} \alpha_1 = \dfrac{\mathrm{d}\omega_1}{\mathrm{d}t} \\ \alpha_2 = \dfrac{\mathrm{d}\omega_2}{\mathrm{d}t} \end{cases}, \begin{cases} a_1 = L_{\mathrm{S}}\alpha_1 \\ a_2 = L_{\mathrm{T}}\alpha_2 \end{cases}, \begin{cases} W_1 = m_{\mathrm{S}}g \\ W_2 = m_{\mathrm{T}}g \end{cases}, \begin{cases} M_1 = I_1\alpha_1 \\ M_3 = I_2\alpha_2 \end{cases}.$$

$$\begin{cases} a_{x1} = a_1\sin\left(\beta-\beta_0\right) \\ a_{y1} = a_1\cos\left(\beta-\beta_0\right) \end{cases}, \begin{cases} a_{x2} = a_2\sin\left(\alpha-\alpha_0\right) \\ a_{y2} = a_2\cos\left(\alpha-\alpha_0\right) \end{cases}.$$

To determine the mass and length of the segments of lower limb links and joint, following data has been used from.[2,3]

1) Mass of a shank (m_1 or m_S) is 4.44% of the total mass of person.

2) Mass of a thigh (m_2 or m_T) is 11.5% of the total mass of person.

3) Length of the shank (L_S) is 26% of the total height of the person.

4) Length of the thigh (L_T) is 24%of the total height of the person.

5) Location of the center of the mass for shank (L'_S) is 43.3% from the start of the ankle.

6) Location of the center of the mass for thigh (L'_T) is 43.3% from the start of the knee.

Accordingly, $F_{xA}^{(1)}$, $F_{yA}^{(1)}$ can be obtained and then calculating the kicking force by the following equation:

$$F = \sqrt{F_{xA}^{(1)2} + F_{yA}^{(1)2}} \ .$$

Video S1. Lower-limb monitoring in dynamic balance training with the R-TENG on the knee joint.

A video shows the R-TENG assisting the quantitative measurement in a dynamic balance training is provided. In this demonstration, the subject imitates the possible postures of a patient with balance disorders before and after rehabilitation exercises. The improvement of the subject's physical capacity and gait/posture stability can be quantitatively measured by the lasting time of the one leg-lifting standing and foot height.

Video S2. A treasure-hunting VR game with the R-TENGs on the hip and knee joints.

Six actions are being demonstrated including two motions (walk and run) and four direction-based motions (right/left shift and right/left turn). The bending movement of the right knee is defined as commands to control the virtual figure to walk or run regarding the bending angles (15 and 60 degrees). For the direction-based motions, the right hip-sensor experiencing the complete kicking forward or backward triggers the left shift/turn or right shift/turn, in which case the rotation degree determines the shift or turn motion (15 and 30 degrees).

Video S3. A football training program with the R-TENGs on the hip and knee joints.

Two kinds of kicking motions are conducted including a straight kick and a bent leg kick. In this training program, the straight kick is set as the command for kicking the football to the right side, while the bent leg kick sets off the football motions to the left area. Based on different kicking force, the distance of the football trajectory in the VR space can be controlled in three schemes.

Reference

[1] R. F. Zernicke, E. M. Roberts, *Med. Sci. Sports* **1978**, *10*, 21.

[2] T. H. Baluch, A. Masood, J. Iqbal, U. Izhar, U. S. Khan, *Int. J. Mech. Aerospace, Ind. Mechatron. Manuf. Eng.* **2012**, *6*, 904.

[3] G. A. Jackson, *Electron. Commun. Eng. J.* **1989**, *1*, 61.
